# Specificity of MYB interactions relies on motifs in ordered and disordered contexts

**DOI:** 10.1093/nar/gkz691

**Published:** 2019-08-10

**Authors:** Peter S Millard, Konrad Weber, Birthe B Kragelund, Meike Burow

**Affiliations:** 1 DynaMo Center, Department of Plant and Environmental Sciences, University of Copenhagen, 1871 Frederiksberg C, Denmark; 2 Copenhagen Plant Science Centre, Department of Plant and Environmental Sciences, University of Copenhagen, 1871 Frederiksberg C, Denmark; 3 Structural Biology and NMR Laboratory, Department of Biology, University of Copenhagen, 2200 Copenhagen, Denmark

## Abstract

Physical interactions between members of the MYB and bHLH transcription factor (TF) families regulate many important biological processes in plants. Not all reported MYB–bHLH interactions can be explained by the known binding sites in the R3 repeat of the MYB DNA-binding domain. Noteworthy, most of the sequence diversity of MYB TFs lies in their non-MYB regions, which contain orphan small subgroup-defining motifs not yet linked to molecular functions. Here, we identified the motif mediating interaction between MYB TFs from subgroup 12 and their bHLH partners. Unlike other known MYB–bHLH interactions, the motif locates to the centre of the predicted disordered non-MYB region. We characterised the core motif, which enabled accurate prediction of previously unknown bHLH-interacting MYB TFs in *Arabidopsis thaliana*, and we confirmed its functional importance *in planta*. Our results indicate a correlation between the MYB–bHLH interaction affinity and the phenotypic output controlled by the TF complex. The identification of an interaction motif outside R3 indicates that MYB–bHLH interactions must have arisen multiple times, independently and suggests many more motifs of functional relevance to be harvested from subgroup-specific studies.

## INTRODUCTION

Transcription factors (TFs) are involved in the regulation of all physiological processes of multicellular organisms from development over metabolism to stress responses. Binary and higher order complexes constitute protein-level regulation of TF activity and hence underlie the functional output; some TFs obligately require homo- or hetero-dimerization for activity (1). These interactions serve to integrate information about e.g. cellular status or signalling events leading to control of the biological processes affected by the TFs. Therefore, specific interactions and tight regulation are critical for accurate TF function and for tight control of cross-talk between different pathways securing signalling fidelity.

**Figure 1.**
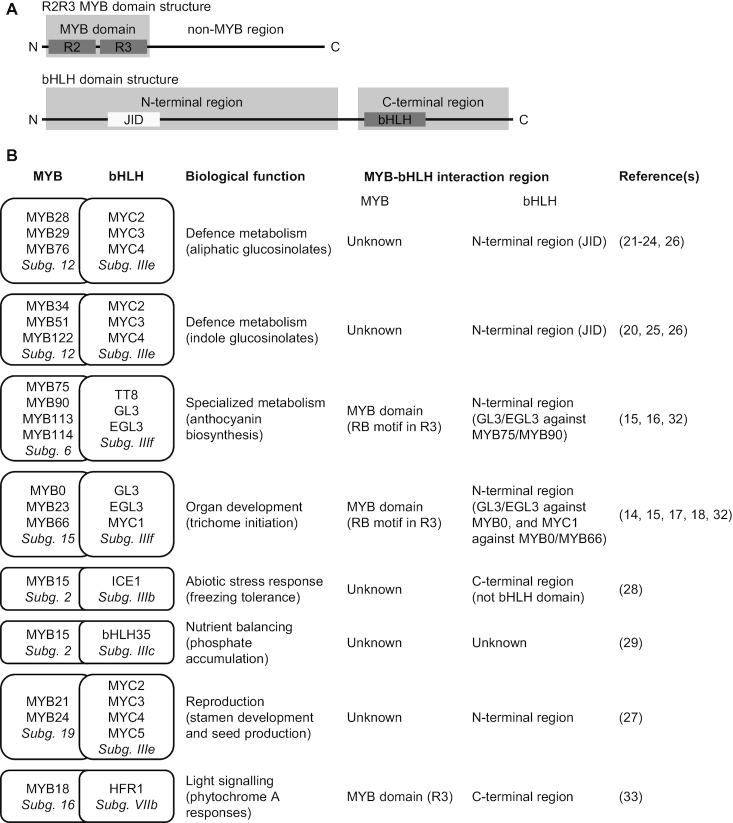
Interactions between MYB and bHLH TFs. (**A**) Typical domain structures of R2R3 MYB and bHLH TFs. The DBD (R2R3 and bHLH) and JAZ interaction domain (JID; not present in all bHLH proteins) are boxed, and larger N- and C-terminal regions of bHLH proteins are indicated to illustrate interaction regions from (**B**). (B) Biological functions regulated by MYB–bHLH interactions, and the interacting regions, when known. The phylogenetic subgroups (Subg.) are indicated in italics (3,4).

In higher plants, MYB and basic helix-loop-helix (bHLH) TFs constitute the two most abundant TF families, each containing more than a hundred members in *Arabidopsis thaliana* (2). Both have been defined from structural properties of their DNA-binding domains (DBD), the MYB and bHLH domains, respectively (Figure 1A). For the MYB domain, the number of MYB repeats varies. In plants, the most common type is the R2R3-type, which contains two repeats most similar to the second and third repeats of vertebrate MYB TFs (3). bHLH domains comprise basic DNA-interacting regions that are connected to amphipathic α-helices enabling homo- and heterodimerization (4,5). Structural information on the remainder of the proteins is extremely sparse, partially because plant TFs in general are predicted to contain extensive disordered regions in their non-DBD regions, likely allowing them to dynamically interact with many different partners (6–9). Both families are divided into phylogenetic subgroups based on conserved sequence motifs in their non-DBD regions (3,4). Each subgroup typically contains 1–3 conserved sequence motifs, many for which the function remains to be established.

Interactions between TFs are common and physical interactions between MYB and bHLH TFs are widespread (10). Complex formation between these two families in response to developmental and environmental cues leads to changes in a large variety of physiological processes (1,2), including organ development, nutrient accumulation, light signalling and various metabolic pathways (Figure 1, and references therein) (3,4,11,12). These physiological processes are associated with distinct TF subgroups.

For some MYB–bHLH interactions, the MYB component of the complex specifies the activity (13). This occurs e.g. when GL3 (from bHLH subgroup IIIf), interacts with MYB75 (from MYB subgroup 6) to control anthocyanin biosynthesis, or when GL3 interacts with MYB0 (from MYB subgroup 15), to regulate trichome initiation (14–18). The same applies to the interaction between subgroup 12 MYB TFs and the subgroup IIIe bHLH TFs MYC2, MYC3 and MYC4, where the MYB component specifies the class of glucosinolates synthesized (aliphatic or indole) (19–26). The same bHLH TFs (MYC2, MYC3 and MYC4 from bHLH subgroup IIIe) are involved in the regulation of stamen development and seed production when they interact with subgroup 19 MYB TFs (27). In both cases, the MYC TFs provide the regulatory context in terms of their activation upon jasmonic acid (JA) signalling (26,27). A case of the opposite scenario is MYB15 (from MYB subgroup 2), which interacts with ICE1 (from bHLH subgroup IIIb) to regulate freezing tolerance (28), but MYB15 is also involved in the regulation of phosphate accumulation when interacting with bHLH35 (from bHLH subgroup IIIc) (29). Possibly, ICE1 additionally plays a role in the regulation of phosphate accumulation by the MYB15-bHLH35 complex (29). In all these examples, phenotypic output is generated through the combinatorial effects of MYB–bHLH complex formation. However, specificity determinants that ensure fidelity and prohibit subgroup cross-talk remain to be uncovered. Of relevance to this, it is important to note the high sequence conservation in the DBDs, versus the non-MYB regions (i.e. outside the DBDs) (3,11). As most of the sequence diversity of MYB TFs lies in the non-MYB regions, these may be more important for functional specialization than so far recognized.

Different MYB–bHLH binding sites must enable highly specific interactions, but for most cases the features responsible for this are not well understood (Figure 1). On the bHLH side of MYB–bHLH complexes, a number of proteins have been investigated, but no detailed information on the binding sites exists. However, it is clear that in some cases the region N-terminal to the bHLH domain is sufficient for interaction (subgroups IIIe and IIIf), whereas in other cases it is the region C-terminal to the bHLH domain (subgroup IIIb). In maize, the N-terminal region of the bHLH TFs R and B mediate interactions with specific solvent-exposed residues in the R3 repeat of their MYB partner protein C1 (30,31). Further work on the Arabidopsis homologues of these MYB and bHLH TFs revealed that on the MYB side, a [DE]Lx_2_[RK]x_3_Lx_6_Lx_3_R motif, contiguously surface-exposed on the structured R3 repeat, mediates those MYB–bHLH interactions (32). However, this motif does not explain all MYB–bHLH interactions. The motif mediates interactions with R/B-like bHLH TFs, defined as bHLH TFs that interact with the WD40 protein TTG1, and thus belong to the same regulatory network (32). In this work, we term it the RB motif.

Since MYB–bHLH complexes regulate so many highly diverse processes, it has been hypothesised that interaction between these two TF families could have a long evolutionary history (2). Further developing this hypothesis, we propose that a distant ancestral interacting pair has given rise to the many interacting pairs we see today. If this is true, contemporary MYB proteins share a bHLH-binding site of the same evolutionary origin, and we must expect similarities in terms of position, sequence, structure or a combination thereof. The RB motif is not present in other MYB TFs interacting in other MYB–bHLH complexes. Yet, other MYB TFs may have motifs that mediate specific interactions with *their* partner bHLH TFs, derived from the same ancestral feature as the RB motif. If partnered MYB–bHLH subgroups co-evolved subgroup-specific interactions from a motif present in the R3 repeat of an ancestral MYB TF, this could explain both the high prevalence and diversity of biological functions regulated by MYB–bHLH interactions. Further, it would allow high specificity and necessary control of cross-talk between regulatory complexes. This hypothesis is supported by the fact that the interaction between MYB18, which lacks an RB motif, and its bHLH partner HFR1 was disrupted by mutation of residue Arg-97 on MYB18, which, like the RB motif, is present in the R3 repeat (33). The currently known bHLH-binding sites in MYB TFs are all located in the R3 repeat of the DBD (Figure 1).

One MYB subgroup of specific interest in terms of MYB–bHLH interaction specificity is subgroup 12, consisting of MYB28, MYB29, MYB76, MYB34, MYB51 and MYB122. The biological importance of interaction with their bHLH partners MYC2, MYC3 and MYC4 is well established, regulating glucosinolate biosynthesis and thereby impacting fitness upon herbivory (26). On the bHLH side, the binding site has been narrowed down to the same region as is responsible for interaction with JAZ repressors, constituted by the JAZ interaction domain (JID), located in the N-terminal domain (26) (Figure 1). The MYC N-terminal domain is structured and a crystal structure of MYC3 has been solved alone and in complex with a JAZ peptide (34). On the MYB side, however, no information on the binding site is available, and MYB TFs from subgroup 12 do not contain an RB motif. Thus, it is completely unexplored how these proteins interact with their bHLH partners.

In this work, we identify the motif responsible for mediating interactions between MYB TFs from subgroup 12 with MYC3 and MYC4. We found that the interaction was not mediated through the R3, or even the DBD, but by one of the conserved motifs located in the centre of the non-MYB region, showing features characteristic of a short linear motif (SLiM), and therefore unlikely to be evolutionarily related to the RB motif. The motif coincides with the subgroup 12 defining motif [L/F]LN[K/R]VA. We were able to define the core residues of the MYC-interaction motif, MIM, to reside on three key positions. Our findings stress the importance of determining molecular functions of subgroup-specific motifs in these protein families.

## MATERIALS AND METHODS

### Cloning

All wild-type (WT) DNA sequences were amplified from cDNA or in-house plasmids, except for *GL3*, the template for which was kindly provided by Ralf Stracke (Bielefeld University). Full-length *MYB29* and *MYB75* encoding altered motifs were ordered as synthetic genes from General Biosystems (NC, USA), shorter mutated *MYB29* versions for MIM characterisation were ordered as gene fragments from Twist Bioscience (CA, USA), full-length *MYB29* encoding proteins with the amino acid residue substitutions L190A or L190V were ordered as synthetic genes from Twist Bioscience, and all were then subcloned into the respective vectors. Constructs for split-ubiquitin assays were generated by amplifying template DNA with primers to add SfiI overhangs compatible with directional cloning into the pFRB (prey) and pFKBP12 (bait) vectors (35). For the expression of *MYB29-WT, MYB29-L190A* and *MYB29-L190V* in *A. thaliana*, the pFRU35S plasmid was generated from a derivative of the P2P3 double Gateway vector (Invitrogen), where the antibiotic selection sequence was replaced by the cassette for selection of transformants by fluorescence microscopy of the pFAST-R05 plasmid (36). The vector was further modified by introducing a USER™ cassette by digestion with SacII and HindIII, followed by ligation to an oligo pair (5′-3′ sequences AGCTTGCTGAGGCTTAATTAAACCTCAGCCCGC and GGGCTGAGGTTTAATTAAGCCTCAGCA). The 35S promoter sequence was amplified from pCambia230035SU (37) using the primer pair 5′-3′ GGTTTAAUTAAGCCTCAGCCTGCAGG and GGCTTAAUTCTAGAGATCCGTCAACATGGTG and inserted into the target vector by USER™ cloning (37). Template DNA was amplified with primers adding a uracil-containing overhang suitable for cloning into the pFRU35S plasmid by USER cloning. For recombinant expression, part of *MYC4* encoding the N-terminal region was amplified with primers that add overhangs containing a C-terminal 6×His-tag and to be suitable for cloning into pET52u by USER cloning (38). All constructs were verified by Sanger sequencing (Macrogen Europe).

### Split-ubiquitin assays

The rapamycin-compatible yeast strain NMY51 *TOR1-1 Δfpr1* (35) was transformed according to DUALhunter™ kit instructions (Dualsystems biotech). Briefly, overnight 2×YPAD cultures were diluted to OD_600_ = 0.2, and incubated at 30°C, 150 RPM for 4–6 h in 2×YPAD, before harvesting the cells by centrifugation at 700 *g* for 5 min. The cells were washed with H_2_O, then 0.1 M LiOAc, before resuspending in 0.1 M LiOAc with 15% glycerol. The competent cells were then aliquoted and stored at −80°C until transformation. For double transformation with bait and prey plasmids, competent cells were thawed on ice, and mixed with transformation mix (240 μl 50% PEG-3350, 35 μl 1 M LiOAc and 25 μl 2 mg/ml ssDNA per transformation) and 200 ng of each plasmid, before being exposed to a 42°C heat shock in a water bath for 45 min. The cells were then chilled on ice for a few minutes, sedimented by centrifugation (3000 *g*, 1 min), resuspended in sterile H_2_O, plated on SD-LW plates and grown at 30°C for 2–3 days.

For drop tests, cells from three individual colonies of each bait/prey combination were grown overnight in liquid SD-LW media. Next day, culture densities were normalized to OD_600_ = 0.05, dilution series (OD_600_ = 0.05, OD_600_ = 0.005, OD_600_ = 0.0005 and OD_600_ = 0.00005) were spotted on square plates (SD-LW, SD-AHLW and SD-AHLW+Rapa) and grown for 2 days at 30°C before taking pictures.

### Generation of transgenic plants

Plants were transformed by floral dip (39). Competent agrobacterium (strain C58, PGV3850) were transformed with pFRU35S plasmids for expression of *MYB29-WT, MYB29-L190A* or *MYB29-L190V* by electroporation, and plated on YEP media containing rifampicin and spectinomycin, and grown at 28°C for 2 days. Liquid YEP media (3 ml) containing rifampicin, spectinomycin and carbenicillin was inoculated with one colony from the transformation plate and grown overnight at 28°C (150 RPM). The 1 ml culture was added to 100 ml liquid YEP media containing rifampicin, spectinomycin and carbenicillin, and grown overnight at 28°C (150 RPM). The cultures were sedimented by centrifugation (4000 *g*, 20 min), and resuspended in 200 ml 5% (w/v) sucrose + 0.05% (v/v) Silwet L-77. *Arabidopsis thaliana* Col-0 *myb29-1* plants (23) were dipped in the Agrobacterium/sucrose/Silwet L-77 solution, and gently agitated for 5 s, before being transferred to the growth chamber in a closed plastic bag. After 24 h, the plastic bag was removed, and the plants were grown for three more weeks before the seeds were collected. Positive seeds were sorted using a Leica M205 FA microscope (excitation filter 546/10, emission band pass filter 600/40), to detect the red fluorescence from the RFP protein expressed under the seed coat-specific OLE1 promoter (36).

### Glucosinolate analysis

Seeds were sown in a randomized block design and stratified at 4°C for 4 days. After stratification, the trays were moved to a chamber with the following settings: 21°C, 80–120 μE/(m^2^*s), 16 h light, 70% relative humidity. A single mature rosette leaf was harvested and weighed from 4-week-old plants, and desulfo glucosinolates were purified and analysed by LC-MS/TQ (alternate protocol 2 as described by (40)), using 5 nmol *p*-OH-benzyl glucosinolate per sample as an internal standard.

### Statistical analysis

Statistical analyses were carried out in RStudio version 1.0.153 (R version 3.4.1). Significant factors and differences between groups were tested with an ANCOVA using the lm function of the stats package. Posthoc testing was performed by Tukey's Honest Significant Differences test (using the HSD.test function of the agricolae package (41), and the default *P*-value cut-off *P* < 0.05) or by pairwise *t*-tests (using the pairwise *t*-test function).

### Transcript analysis

Immediately prior to harvesting leaves for glucosinolate analysis, single mature rosette leaves were harvested, flash frozen in liquid nitrogen and stored at −80°C until transcript analysis was performed. Total RNA was extracted from frozen leaf material homogenized to fine powder in RETSCH bead-mill. RNA was extracted from tissue powder using HTP96 protocol as described in (42). Native RNA quality was assessed by electrophoresis on a 1% agarose gel. Subsequently, 1 μg of total RNA was DNase1 (Sigma) treated and used for cDNA synthesis with a blend of random hexamers and oligo(dT) primers in a 20 μl reaction using iScript™ kit (Biorad). The obtained cDNA was diluted 1:10 in water and 2 μl was used in a 10 μl SYBR green (PowerUp SYBR™ Applied Biosystems) quantitative polymerase chain reaction (qPCR) reaction, run in a CFX384 Touch™ real-time PCR detection system (Biorad). qPCR of *MYB29* (At5g07690) and the reference transcript *UBC21* (At5g25760) were performed in standard cycling mode condition according to SYBR green manufacturer. Three technical replicates were run for each biological replicate. Relative quantification was calculated with kinetic PCR efficiency correction and normalized to *UBC21* and further to *MYB29* expression level in Col-0. *MYB29* primers (5′-3′): GAACACGCATCTCAAAAAGCTCCTG and ACTTTGGAGAGATGGAACCCGATTG. *UBC21* primers (5′-3′): CTGAGCCGGACAGTCCTCTTAACTG and CGGCGAGGCGTGTATACATTTGTG.

### Recombinant protein expression and purification

Single colonies of freshly transformed *Escherichia coli* BL21(DE3), carrying the pET52u plasmid for expressing MYC4 N-terminal domain (AA55-253; MYC4Nt) with a C-terminal 6×His tag, were inoculated in LB with ampicillin and grown overnight (37°C, 200 RPM). The next day, the culture was diluted to OD_600_ = 0.2 with cold (4°C) LB with ampicillin and grown at 18°C (100 RPM) in a baffled Erlenmeyer flask until OD_600_ = 1.0 (∼7.5 h). Protein expression was induced by addition of 0.1 mM IPTG, and the cultures grew for further 18 h at 18°C (100 RPM), before harvesting the cells by centrifugation (4000 *g*, 20 min), and storing the bacterial pellet at −20°C until protein extraction and purification. For extraction and purification, the bacterial pellet was resuspended in BugBuster Master Mix (Merck Millipore), 2.5 ml per 50 ml bacterial culture, containing 1× ethylenediaminetetraacetic acid-free complete protease inhibitor cocktail (Roche) and 1 mM dithiothreitol (DTT). Lysis proceeded by slow end-over-end rotation at room temperature for 30 min, and the lysate was cleared by centrifugation (20.000 *g*, 20 min, 4°C). The supernatant was diluted five times with His binding buffer (20 mM sodium phosphate, 500 mM NaCl, 40 mM imidazole, 1 mM DTT, pH 7.4) and filtered through a 0.2-micron filter using a syringe, before applying to a HisTrap HP 5 ml (GE Healthcare) IMAC column charged with Ni(II)Cl_2_. After washing thoroughly, bound protein was eluted with a linear buffer gradient to elution buffer (His binding buffer with 500 mM imidazole) over 20 column volumes, and 1.9 ml fractions collected in a 96-deepwell plate. Based on absorbance at 280 nm during elution, fractions were selected for analysis by sodium dodecyl sulphate-polyacrylamide gel electrophoresis and those fractions showing only the band of the recombinant protein at 22.7 kDa were pooled and concentrated using 3-kDa cut-off PES membrane ultrafiltration centrifugal tubes (Thermo Scientific™ Pierce). Concentrated protein was centrifuged at 20 000 *g*, 4°C for 10 min before being dialysed overnight to PBS pH 7.4 with 1 mM DTT. Protein concentration was estimated using the Lambert–Beer law based on absorbance at 280 nm, after blanking with the dialysis buffer (dialysate) and protein was stored in aliquots at −80°C. Aliquots of dialysate were stored at −20°C.

### Bio-layer interferometry

Biotinylated peptides were synthesized by TAG Copenhagen A/S (Copenhagen, Denmark). Lyophilized peptide was resuspended in H_2_O to a concentration of 400–500 μM and stored in aliquots at −80°C. On the day of experiment, 0.05% bovine serum albumin was dissolved in PBS pH 7.4 with 1 mM DTT (freshly thawed dialysate), to use as the assay buffer. Peptide and protein were freshly thawed on ice and diluted with assay buffer. The final concentration used was 1 μM for each peptide, and a dilution series of MYC4Nt ranging from 130 to 0.1 μM. Binding was measured with a BLItz Bio-Layer Interferometer (Pall ForteBio) using the advanced kinetics program in the BLItz Pro 1.2 software. Referenced sensorgrams were fitted locally using a 1:1 model with the BLItz Pro 1.2 software, to extract equilibrium response values. Equilibrium response values were plotted against concentration in OriginPro 2016 and fitted to a Hill equation to determine the equilibrium dissociation constant.

## RESULTS

### MYB–bHLH interactions are mediated by different MYB regions

**Figure 2.**
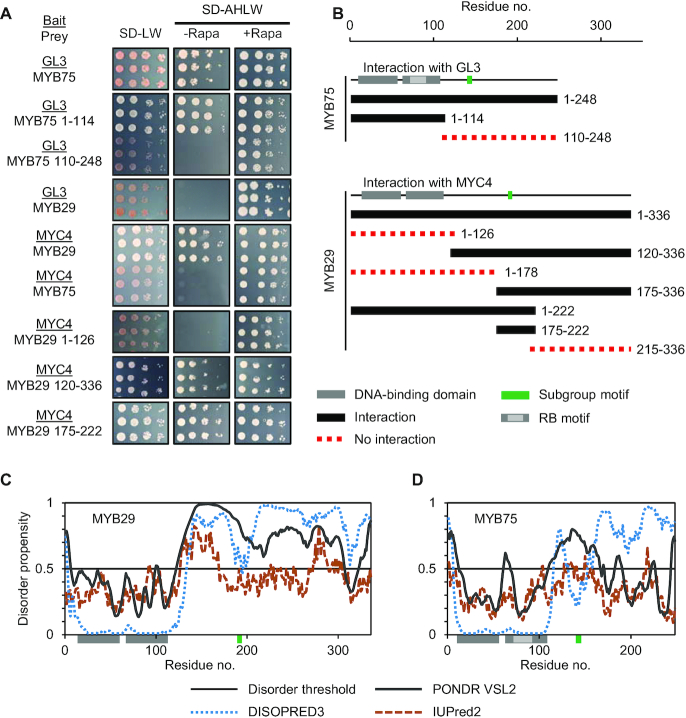
Identification of a new bHLH interaction interface in plant R2R3 MYB TFs. (**A**) Split-ubiquitin assays with full-length bHLH proteins (GL3 and MYC4) as baits (underlined) against full-length or truncated MYB proteins (MYB75 and MYB29). Cultures from three independent colonies were spotted on SD-LW (positive control for culture density and viability), SD-AHLW (test for interaction) and SD-AHLW+Rapa (positive control for ability of bait and prey fusion proteins to interact (35)). (**B**) Schematic diagram indicating truncated versions of MYB75 that interact with GL3, as previously reported (32), and truncated versions of MYB29 that interact with MYC4. The location of the DBD is shown in grey, conserved subgroup 6 and subgroup 12 motifs are shown in green, and the RB motif mediating interaction between MYB and R/B-like bHLH TFs is shown in light grey (32). Constructs marked in black interact, whilst those displayed as a red dotted line do not interact (see also Supplementary Figures S1 and 2). (**C** and **D**) Sequence specific disorder predictions of (C) MYB29 and (D) MYB75 using DISOPRED3 (45), PONDR VSL2 (43,44) and IUPred2 (46).

To identify interaction sites mediating specific MYB–MYC complex formation, we selected two TFs from each family. These were MYB28 and MYB29 from MYB subgroup 12, and two of their partner bHLH TFs, MYC3 and MYC4. To define the region mediating the interactions we applied a chemically inducible dimerization split-ubiquitin system (35). Split-ubiquitin systems are superior to classical yeast-two-hybrid assays as they permit activation domain-containing bait proteins, and full-length TFs can thus be investigated. As controls, we included the interaction between GL3 and MYB75, where a fragment comprising the MYB DBD (residues M1-P114) has been shown to be sufficient for this interaction, whereas the non-MYB region (residues K110-D248) was unable to interact (32). Co-expressing the interacting bait/prey combination GL3-MYB75 (either full-length MYB75 or the DBD), results in growth on SD-AHLW plates, whereas co-expressing the GL3 bait with a prey comprising the non-MYB region of MYB75 does not (Figure 2A). Addition of rapamycin forces the interaction between bait and prey fusion proteins (35), as seen for the combination of GL3 with the non-interacting non-MYB region of MYB75. This is used as an in-built positive control for correct expression and functionality of the prey fusion protein and thereby reduces the incidence of false negatives.

We next proceeded to narrow down the region mediating the interaction between MYB28/MYB29 and MYC3/MYC4. Like for MYB75, we divided MYB29 into the DBD (residues M1-H126) and the non-MYB region (G120-I336). Here we observed that the DBD was neither sufficient, nor necessary for the interaction, which instead was mediated by the non-MYB region (Figure 2A). We then tested consecutively smaller overlapping protein fragments from the non-MYB region with a minimal length of 35 residues. The fragments were designed to avoid fragmentation of regions conserved between MYB28 and MYB29. The results showed that a 47-residue fragment of MYB29 (S175-L222) was sufficient for the interaction (Figure 2A and B). We tested several additional truncated versions for both MYB28 and MYB29 against both MYC3 and MYC4 (Supplementary Figures S1 and 2), and all supported the same region of the proteins as being required for the interactions; a region that contained the subgroup 12-defining motif, [L/F]LN[K/R]VA (Figure 2B).

After having established that a feature located far outside the DBD mediates the interaction, we addressed the structural properties of the non-MYB region. Several web servers (43–46) predicted that the DBDs of both MYB29 and MYB75 are globular, structured domains (Figure 2C). In contrast, and by the same servers, the remainder of the proteins, which showed very low sequence conservation, displayed large stretches predicted to be intrinsically disordered (Figure 2C); a feature of plant TF non-DBD regions that has been discussed previously (6,7). The RB motif locates to surface-exposed residues in the structured R3 repeat of the DBD (32), whereas the new binding site identified here locates to a disordered region outside the MYB domain (Figure 2C).

### A conserved motif mediates interactions between MYB TFs from subgroup 12 and their bHLH interaction partners

Whenever the MYB subgroup 12 motif, [L/F]LN[K/R]VA, was part of the MYB28 or MYB29 protein fragment tested, we observed an interaction (Figure 2B; Supplementary Figures S1 and 2). Since this motif is present in all six MYB TFs from subgroup 12, and since they have all been shown to interact with MYC2, MYC3 and MYC4 (25,26) we hypothesised that this could be the motif mediating interaction. To test this, we introduced mutations to the motif. As a control, we again turned to the known interaction between MYB75 and GL3, which is mediated through the RB motif in the R3 repeat of MYB75. As MYB TFs from subgroup 12 interact specifically with MYC3 and MYC4, but not GL3 and MYB75 from subgroup 6 interacts specifically with GL3, but not MYC3 and MYC4, we aligned the protein sequences of MYB75 and MYB29 (Supplementary Figure S3) to identify residues of interest to mutate to abolish MYC interaction in MYB29, and GL3 interaction in MYB75. The RB motif is present in a structured and conserved part of the protein, the DBD, which facilitates sequence alignment, whereas the subgroup 12 motif is present in the non-MYB region, where sequence conservation is low, and sequence alignment challenging. We identified a region in MYB75 (_134_LKNNVY_139_) and introduced three mutations to convert the subgroup 12 motif to the corresponding sequence of MYB75.

**Figure 3.**
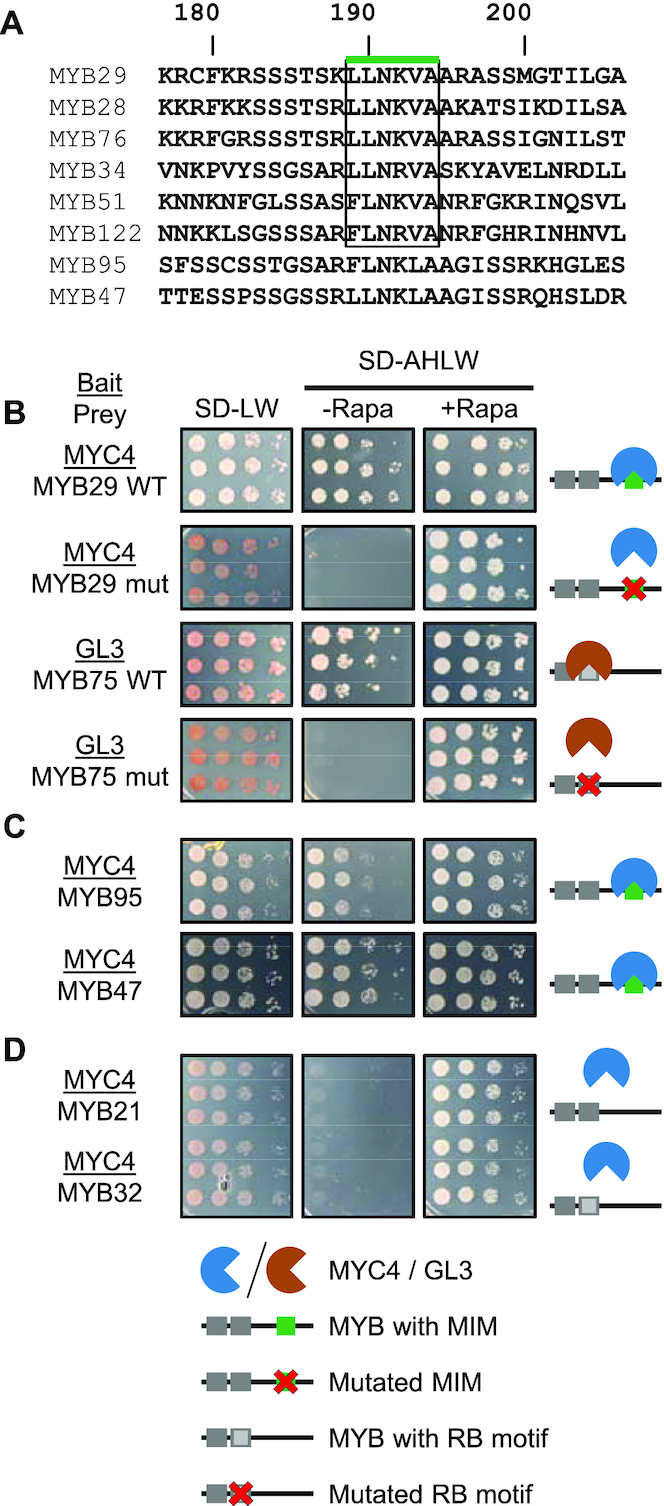
The MYB subgroup 12 motif is the MYC-interaction motif (MIM) and accurately predicts novel interactions. (**A**) Alignment of MIM-containing MYB TFs from *Arabidopsis thaliana*, with numbering according to MYB29. The subgroup 12 motif is enclosed by a box marked with a green line. (**B**–**D**) Split-ubiquitin assays with MYC4 or GL3 as baits (underlined) against (B) WT and mutated (mut) versions of MYB29 or MYB75, (C) MYB95 and MYB47, and (D) MYB21 and MYB32. In (B), the mutations to abolish the MIM are L190K, K192N and A194Y. The RB motif of MYB75 was mutated to the corresponding residues in the MYB29 R3 repeat (DLLLRLHRLLGNRWSLIAGR to QILLMLHRSLGNRWSVIAGH; motif residues underlined, as the motif is [DE]Lx_2_[RK]x_3_Lx_6_Lx_3_R (32).

Mutating residues of the RB motif in MYB75 to the corresponding residues of MYB29 abolished interaction with GL3, as expected (Figure 3B). Similarly, mutating residues of the subgroup 12 motif in MYB29 to the corresponding residues of MYB75 (according to the alignment mentioned above) abolished interaction with MYC3 and MYC4, confirming that the subgroup 12 motif is the MYC-interaction motif, [L/F]LN[K/R]VA, referred to here on as MIM (Figure 3B and Supplementary Figure S4). With four additional constructs, we tested whether the RB motif and the MIM were sufficient to mediate interaction in a non-native context. By mutating residues in the R3 repeat of MYB29 to the corresponding residues of MYB75, we introduced a putative RB motif, and by mutating residues in the MYB75 non-MYB region to the corresponding residues of MYB29, we introduced a putative MIM. Co-expressing these proteins with GL3, MYC3 and MYC4, we found that the motifs were not sufficient for interaction by themselves, as mutating residues in MYB75 to create a MIM, or mutating residues in MYB29 to create a RB motif did not result in any new interactions (Supplementary Figure S4). Still, in subgroup 12 MYB TFs, the MIM mediates interaction with MYC3 and MYC4, as site-specific mutagenesis abolished the interactions (Figure 3B).

### Presence of the MIM accurately predicts new MYB–bHLH interactions

Having defined the MIM, we used a bioinformatics approach to identify other proteins in *A. thaliana* containing a MIM. Based on the conservation of the motif (Figure 3A), we allowed some flexibility at positions 1 and 2 ([LVFI]), 4 ([KR]) and 5 ([LVFI]), and included a positively charged residue ([KR]) at position 8. Thus, as query we used sequences of subgroup 12 MYB TFs, and conducted a PHI-BLAST with the PHI pattern [LVFI]-[LVFI]-N-[KR]-[IFLV]-A-X-[KR] against all *A. thaliana* proteins. Since hydrophilic residues, especially Ser, are conserved N-terminal to the MIM, we also looked for this feature in the BLAST results. Interestingly, apart from its consensus MIM, _153_LLNRVA_158_, MYB34 from subgroup 12 contains an additional putative MIM, _197_LLNKMA_202_, suggesting that it might have multiple interaction motifs. Our search further revealed two R2R3 MYB TFs, MYB95 and MYB47 (Figure 3A), which have not been assigned to a subgroup (3,11). Near the middle of their non-MYB region, similar to the location of the MIM in the subgroup 12 MYB TFs, they contain the sequences _167_FLNKLA_172_ (MYB95) or _165_LLNKLA_170_ (MYB47), which are similar to the MIM except for the Leu at the fifth position, being a Val in all subgroup 12 MYB TFs. Since the side chains of Leu and Val have similar, hydrophobic chemical properties, we tested whether MYB95 and MYB47 could interact with MYC3/MYC4, and indeed we could confirm those interactions (Figure 3C and Supplementary Figure S4).

Like the MIM-containing MYB TFs examined above, MYB TFs from subgroup 19 (e.g. MYB21 and MYB24) also interact with MYC TFs (Figure 1B) (27). A sequence search showed that they do not contain the MIM identified here, and we therefore tested which region was mediating their MYC-interaction. However, in our split-ubiquitin system, we were not able to reproduce the interaction between MYB21 and MYC3/MYC4 (Figure 3D and Supplementary Figure S4). We also tested the interaction between MYC3/MYC4 and MYB32, MYB51 and MYB113 (Figure 3D and Supplementary Figure S4). MYB51 (also from subgroup 12 and containing a MIM) interacted with both MYC3 and MYC4, whilst MYB32 and MYB113 (from subgroups 4 and 6, respectively, neither containing a putative MIM) did not. These results further demonstrate that MYB–bHLH interactions are highly specific and that the MIM is responsible for this specific function within a subset of MYB TFs, including those from subgroup 12.

Combining the finding that MYB95 and MYB47 contain a functional MIM, with the MIM present in subgroup 12 MYB TFs, we arrived at the following MIM sequence: [L/F]LN[K/R][V/L]A. Furthermore, our data suggests that MYB95 and MYB47 belong to subgroup 12, or at least share subgroup 12 properties. We next set out to characterise the motif in more detail and determine which residues are critical for interaction.

### Three core residues constitute the MIM

In MYB29, the MIM constitutes residues _189_LLNKVA_194_. To determine which of these residues are strictly necessary for the interaction, we designed a series of variants encoding MYB29 (G120-L222) with different mutations in the MIM and examined their ability to interact with both MYC3 and MYC4. The WT MYB29 G120-L222 interacted with both MYC3 and MYC4 (Supplementary Figures S5 and 6A).

**Figure 4.**
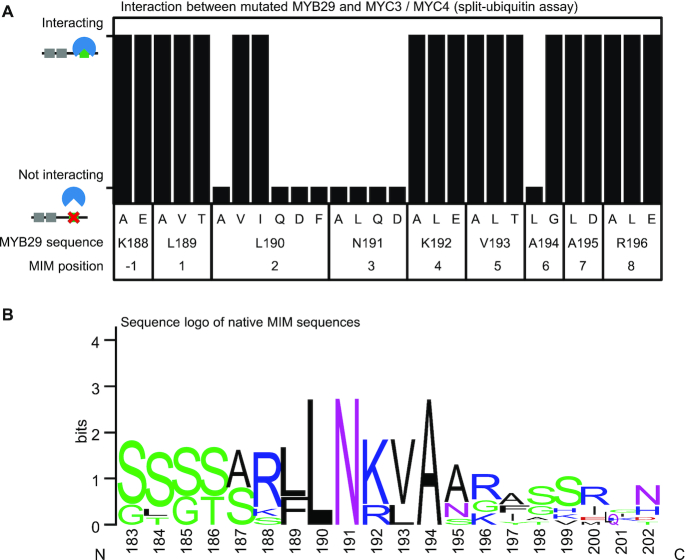
Decomposition of MIM core residues. (**A**) Interaction between MYC3 or MYC4 as bait against mutated versions of MYB29 (G120-L222). Results from split-ubiquitin assays are displayed as tall and short bars indicating interaction and no interaction, respectively. Results from the split-ubiquitin assays for each mutant (including combinatorial mutants) against MYC3 and MYC4 can be found in Supplementary Figures S5 and 6. (**B**) Sequence logo of MIM sequences in *Arabidopsis thaliana* MYB TFs (the eight aligned sequences in Figure 3A), numbered according to MYB29, generated by WebLogo (47).

First, we challenged the importance of each individual side-chain from position −1 to +8, L189 constituting residue 1, (_188_KLLNKVAAR_196_) by a simple alanine scan, or in the case of Ala, by changing it to Gly. Only mutations at positions 2 and 3, L190A and N191A, abolished the interaction (Figure 4A; Supplementary Figures S5 and 6A), which means these two residues constitute core positions of the motif, likely making direct contacts to MYC3/MYC4. We investigated several other substitutions at these two positions, and found that hydrophobic residues (Leu, Val and Ile) were allowed at position 2 (L190_MYB29_), but only Asn was permitted at position 3 (N191_MYB29_) (Figure 4A; Supplementary Figures S5 and 6B). Based on our experiments, MYC4 showed slightly higher promiscuity than MYC3, also weakly interacting when position 2 was mutated to Phe (L190F), or when position 3 was mutated to Gln (N191Q) or Leu (N191L).

Second, based on the alignment in Figure 3A (sequence logo Figure 4B), we formulated several hypotheses about which chemical properties of the other residues in the MIM could be essential for the interaction, and proceeded to test these one by one. In and around the motif there are several positively charged residues (Arg and Lys), and rarely negatively charged ones (Asp and Glu). Thus, positive charges may be important to match the binding partner. We tested this by mutating one, two, or all three positive residues (K188, K192 and R196 at positions −1, 4 and 8) to Glu. However, none of these positions, even when mutated at the same time, abolished the interaction (Figure 4A; Supplementary Figures S5 and 6C).

Mutating position 6 (A194G), where Ala was conserved, did not abolish the interaction, suggesting a residue with a small side-chain is required here. Instead, substitution to Leu (A194L) abolished the interaction with both MYC3 and MYC4 (Figure 4A; Supplementary Figures S5 and 6D), confirming a space requirement at this position.

Both position 1 (L189) and position 5 (V193) could be individually substituted to Ala. As these two positions are conserved as hydrophobic residues (Figures 3A and 4B), there may exist a preference for hydrophobicity. Therefore, those positions were changed to Thr, both individually and in concert, but none of these changes abolished the interaction with either MYC3 or MYC4 (Figure 4A,; Supplementary Figures S5 and 6D), suggesting more promiscuity.

Comparing the results of our mutational analysis of the MIM (Figure 4A) with a sequence logo generated by WebLogo (47) (Figure 4B), we find that mutations at positions 2, 3 and 6 (L190, N191 and A194 in MYB29) abolish interaction, coinciding with the most conserved positions. We therefore conclude that positions 2, 3 and 6 are core positions of the motif, and likely directly involved in the interaction interface.

### MYB29 without a functional MIM is unable to rescue *myb29-1* mutants

MYB TFs from subgroup 12 along with MYC2, MYC3 and MYC4 control biosynthesis of glucosinolates. The *myb28-1 myb29-1* double mutant is almost devoid of methionine-derived aliphatic glucosinolates (23,24,48,49), whilst the *myb34 myb51 myb122* triple mutant is devoid of tryptophan-derived indole glucosinolates (25,26). Further, the *myc2 myc3 myc4* triple mutant is devoid of both aliphatic and indole glucosinolates (25,26). Together with the findings that all six MYB TFs from subgroup 12 can interact with MYC2, MYC3 and MYC4 (25,26), it has been suggested that MYB–MYC interactions are essential for activation of the glucosinolate biosynthetic genes and thus glucosinolate biosynthesis (19). To address the biological relevance of the identified MIM responsible for mediating these interactions, we attempted to rescue the *myb29-1* knockout with the *MYB29* gene, carrying mutations in the MIM coding sequence. We generated transgenic lines containing either *pro35S:MYB29-WT, pro35S:MYB29-L190A* or *pro35S:MYB29-L190V* in the *myb29-1* background. The glucosinolate phenotype of *myb29-1* is a 30% reduction in short chain aliphatic glucosinolates (21,23,24). As the single substitution L190A completely abolished interaction with MYC3 and MYC4 in our split-ubiquitin assay, we expected this construct to be unable to rescue the *myb29-1* phenotype, if the interaction with MYC2, MYC3 and MYC4 via the MIM is essential. Since MYB29 carrying the L190V mutation can interact in our assay, we expected that this construct should be able to rescue the phenotype.

**Figure 5.**
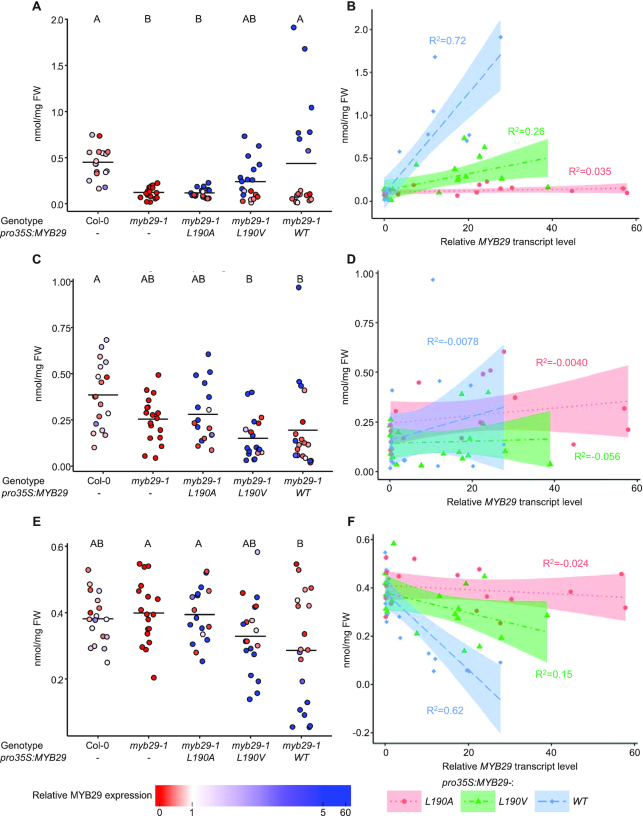
Leaf glucosinolate levels in Col-0, *myb29-1*, or *myb29-1* transgenic plants expressing *pro35S:MYB29-L190A, pro35S:MYB29-L190V* or *pro35S:MYB29-WT* shown in nmol/mg fresh weight of short chain aliphatic (**A** and **B**), long chain aliphatic (**C** and **D**) and indole (**E** and **F**) glucosinolates. A, C, E: Data are grouped by genotype/construct. Fill colours indicate relative *MYB29* transcript levels. Horizontal lines show the mean of each group. Letters above panels A, C and E indicate significant differences (*P*< 0.05) between group means (Tukey's Honest Significant Differences test). B, D, F: Glucosinolate levels were plotted against relative *MYB29* transcript levels. Dotted lines and surrounding shaded areas show linear regressions and the 95% confidence intervals, respectively. Adjusted *R*^2^ is shown in the same colour as the fit. Statistical differences between slopes were tested with an ANCOVA (Table 1 and Supplementary Table S1). Individual glucosinolate levels reported in Supplementary Table S2.

For each of the three transgenes investigated, we analysed leaf glucosinolates and relative *MYB29* expression levels in 17–19 independent T1 plants positive for the selection marker, and in 18 plants from grown-along Col-0 and *myb29-1* plants (Figure 5A–F). Whilst the *myb29-1* mutant accumulated less short chain aliphatic glucosinolates compared to Col-0 (Figure 5A), the levels of long chain aliphatic (Figure 5C) or indole (Figure 5E) glucosinolates were not significantly different. *myb29-1* plants expressing *pro35S:MYB29-L190A* had glucosinolate profiles indistinguishable from *myb29-1*, even for individuals with >50-fold higher *MYB29* expression compared to Col-0 (Figure 5A–F), showing that MYB29 with this single residue mutation is unable to rescue the phenotype even at very high-expression levels.

In contrast, in *myb29-1* plants expressing *pro35S:MYB29-WT*, glucosinolate levels correlated with the expression level of *MYB29*. Plants with high *MYB29* expression showed increased levels of short chain aliphatic glucosinolates (adj. *R*^2^ = 0.72) (Figure 5B), decreased levels of indole glucosinolates (adj. *R*^2^ = 0.62) (Figure 5F), and unchanged levels of long chain aliphatic glucosinolates (Figure 5D). A similar relationship between glucosinolate levels and *MYB29* expression level was observed for *myb29-1* plants expressing *pro35S:MYB29-L190V*, although more modest (adj. *R*^2^ = 0.26 for short chain aliphatic glucosinolates and adj. *R*^2^ = 0.15 for indole glucosinolates). Statistical analysis showed that the overall means of short chain aliphatic glucosinolates were similar between Col-0 and *myb29-1* plants expressing *pro35S:MYB29-WT* or *pro35S:MYB29-L190V* (Figure 5A and Supplementary Table S1). These results demonstrate the ability of the WT and the L190V mutant version of MYB29 to rescue the *myb29-1* phenotype, in accordance with our expectations based on their ability to interact with MYC TFs.

Comparison of the slopes correlating glucosinolate accumulation to *MYB29* expression level showed that *MYB29* expression in plants with the *pro35S:MYB29-L190A* transgene had no effect on the accumulation of either short chain aliphatic, long chain aliphatic or indole glucosinolates (Figure 5B, D and F; Table 1). Conversely, plants with the *pro35S:MYB29-L190V* transgene or the *pro35S:MYB29-WT* transgene had correlation slopes significantly different from 0 for both short chain aliphatic and indole glucosinolates. Further, the correlation slopes of plants with the WT version of MYB29 were significantly steeper than the correlation slopes of plants with the L190V version. This clearly shows that although both constructs were able to rescue the *myb29-1* phenotype when expression levels were high enough (Figure 5A, C and E), *MYB29-WT* increased short chain aliphatic glucosinolate levels more than *MYB29-L190V* at similar expression levels (Figure 5B, D and F). Based on these observations, we hypothesised that the L190V mutation decreased the affinity of the MYC TF interaction, leading to a lower activity by interfering with complex formation. To test this, we next turned to an *in vitro* assay, to gain insight into the relative interaction strengths of the MIM and mutated MIMs for the MYC TFs.

**Table 1.** Comparison of slopes (nmol/mg glucosinolate as a function of relative *MYB29* transcript level) when expressing *pro35S:MYB29-L190A, pro35S:MYB29-L190V* and *pro35S:MYB29-WT* in *myb29-1* background.

Short chain aliphatic glucosinolates				
*pro35S:MYB29*	p (≠0)	p (≠*L190A*)	p (≠*L190V*)	p (≠*WT*)
*L190A*	0.77		0.077	6.3e-12***
*L190V*	0.025*	0.077		1.8e-8***
*WT*	2.0e-13***	6.3e-12***	1.8e-8***	
Long chain aliphatic glucosinolates				
*pro35S:MYB29*	p (≠0)	p (≠*L190A*)	p (≠*L190V*)	p (≠*WT*)
*L190A*	0.40		0.74	0.45
*L190V*	0.89	0.74		0.37
*WT*	0.22	0.45	0.37	
Indole glucosinolates				
*pro35S:MYB29*	p (≠0)	p (≠*L190A*)	p (≠*L190V*)	p (≠*WT*)
*L190A*	0.50		0.16	5.4e-6***
*L190V*	0.038*	0.16		9.0e-4***
*WT*	3.2e-7***	5.4e-6***	9.0e-4***	

Transcript normalized to Col-0. *P*-values refer to significant difference to 0 (≠0) or to other transgenes. The full ANCOVA table can be found in Supplementary Table S1. Significance codes: *P*< 0.001***, *P*< 0.01**, *P*< 0.05*.

### *In vitro* MIM–MYC interaction suggests a correlation between interaction affinity and functional output

To simultaneously address whether the MIM mediates interaction with MYC TFs *in vitro*, whether it interacts directly with the JID and whether differences in binding affinity can explain the different degrees to which MYB29-WT and MYB29-L190V increased glucosinolate accumulation *in planta*, we used bio-layer interferometry. The N-terminal domain of MYC2, MYC3 and MYC4, contains the JID (Figure 1A), which mediates interactions with MYB TFs from subgroup 12 (26). Therefore, we optimized a purification strategy for the MYC4 N-terminal domain (L55-N253; MYC4Nt) using a C-terminal 6×His tag, and designed three biotinylated peptides comprising the WT-MIM, the L190A-MIM and the L190V-MIM, each consisting of 22 residues (F180-G201 of MYB29).

**Figure 6.**
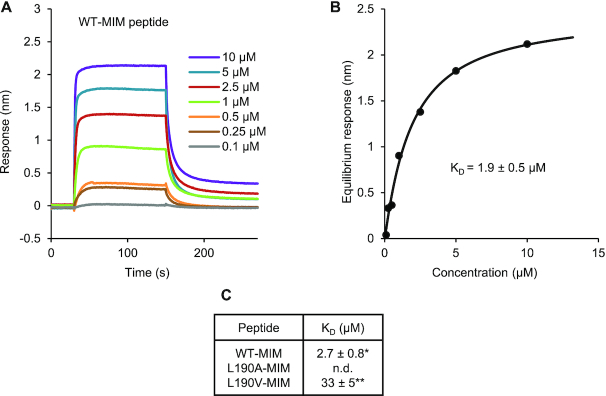
The MIM peptide interacts with MYC4Nt *in vitro*, and the affinity is affected by mutation of L190. N-terminally biotinylated 22-residue peptides comprising the WT (**A** and **B**), L190A or L190V (Supplementary Figure S7) MIM sequences of MYB29 were immobilized on streptavidin biosensors and the binding of a dilution series of MYC4Nt (L55-N253 with C-terminal 6×His tag) was detected. (A) Referenced sensorgrams. (B) Fitting of equilibrium responses for WT-MIM to a Hill equation, including the equilibrium dissociation constant (*K*_D_) ± standard error of the fit. (**C**) *K*_D_ values obtained for WT-MIM, L190A-MIM and L190V-MIM peptides. n.d.: Not determined due to lack of convergence. *Mean ± s.e.m. (of *n* = 2 independent rounds of protein expression). **K_D_ from one fit ± standard error of the fit.

After immobilising biotinylated peptides to streptavidin biosensors, binding of MYC4Nt at various concentrations was recorded. When the WT-MIM peptide was immobilized, binding of MYC4Nt was observed even at sub micro molar concentrations. Fitting of equilibrium responses to a Hill equation resulted in a dissociation constant *K*_D_ = 2.7 ± 0.8 μM (Figure 6), similar to previously reported for MYB–bHLH interactions (50) and fully in line with typical SLiM-based, biologically relevant interactions (51–54). With the L190A-MIM peptide, an extremely weak response was seen only at high (>100 μM) concentrations of MYC4Nt (Supplementary Figure S7). This weak and unstable response is likely an artefact, perhaps resulting from crowding and rearrangement of protein on the biosensor tip, inflicted by the high concentrations used. We attempted to fit the equilibrium responses to a Hill equation, but the fit did not converge. When the L190V-MIM peptide was immobilised, we observed a strong binding signal, but at higher concentrations of MYC4Nt (>10 μM), than was necessary for the WT-MIM (Figure 6 and Supplementary Figure S7). At the highest concentrations of MYC4Nt used (100 and 130 μM), we encountered problems with signal stability as with the L190A-MIM peptide and curve fitting and subsequent calculation of the dissociation constant was therefore not as reliable as with the WT-MIM peptide. The estimated affinity of L190V-MIM for MYC4Nt was ∼10-fold weaker than the affinity of the WT-MIM peptide for MYC4Nt.

These results clearly show that the MIM mediates interaction *in vitro* and that it interacts directly with the MYC4 N-terminal domain. The data obtained from the bio-layer interferometry experiments further established a stronger binding of the WT-MIM peptide compared to that of the L190V-MIM peptide. In contrast, we were unable to detect binding of the L190A-MIM peptide, in accordance with our findings using the split-ubiquitin assay, where the L190A substituted variant was unable to interact with either MYC3 or MYC4 (Figure 4A; Supplementary Figures S5 and 6). The differences in MYC affinity observed for the MIM variants indicate that MYB–MYC interaction kinetics affect the functional output of these TFs *in planta*.

## DISCUSSION

### Multiple independent evolution events facilitated MYB–bHLH interactions

**Figure 7.**
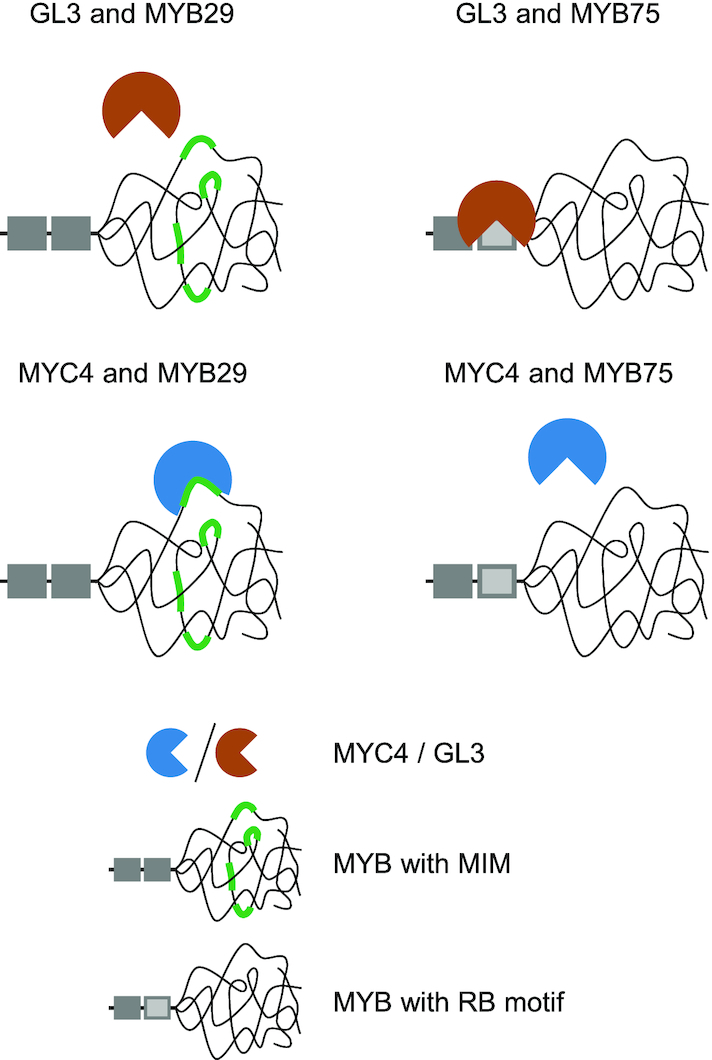
Specificity of MYB–bHLH interactions relies on context dependent motifs. The RB motif, which mediates interaction between e.g. MYB TFs from subgroups 4, 5, 6 and 15 and R/B-like bHLH TFs, constitute surface-exposed residues in a globular domain (32), whilst the MIM identified in this work resides within the predicted disordered non-MYB region.

Interactions between MYB and bHLH TFs regulate various important biological processes in higher plants (Figure 1B). Based on their functional importance and prevalence, we expected MYB–bHLH interactions to be of shared ancestry, which would imply that they adhere to a common mechanism of complex formation. In this work, we discovered the MIM as the motif responsible for the interaction between MYB TFs from subgroup 12 and their bHLH interaction partners, MYC3 and MYC4 (Figures 2 and 3B). We confirmed our findings by (i) successfully predicting previously unknown MYB–bHLH interactions (Figure 3C), (ii) validating the importance of the MIM *in planta* (Figure 5) and (iii) demonstrating interaction between a synthetic peptide comprising the MIM and the purified N-terminal domain of MYC4 from a recombinant source (Figure 6). The combined results from our *in planta* validation and *in vitro* interaction assay show a correlation between interaction affinity and phenotypic output (glucosinolate accumulation) of the TF complex. The structural context of the MIM is very different from a previously characterised motif mediating MYB–bHLH interactions, the RB motif (32). The MIM is not located within a structured domain, but instead resides in the middle of a large predicted intrinsically disordered region (Figure 7). This indicates that the MIM and the RB motif are highly unlikely to have a shared ancestry, which leads us to reject the hypothesis that contemporary MYB–bHLH interacting pairs evolved from a single ancestral interacting pair. Instead, our findings suggest that MYB–bHLH interactions have evolved multiple times, by convergent steps. Such convergent evolution is consistent with one view of evolution of SLiMs, which because of the limited number of core required sequence positions, have been suggested to be able to appear *ex nihilo* (51,55).

### Specificity of distinct motifs mediating MYB–bHLH interactions

With our split-ubiquitin assay, we tested whether either the motif alone (the RB motif or the MIM) outside of its context, was sufficient for the interaction, by introducing the RB motif in MYB29 and the MIM in MYB75. This should change the interaction preferences of MYB29 and MYB75 and introduce new MYB–bHLH interactions (with GL3 and MYC3/MYC4, respectively). Whilst we successfully abolished interactions by targeted mutagenesis, newly introduced motifs did not mediate interaction in non-native contexts (Figure 3 and Supplementary Figure S4). This indicates that even though we understand the primary determinants necessary for interaction (the core motifs), there must be other critical factors we do not yet understand, i.e. the contribution of residues in the flanking regions, the local chemical environment, the disordered structural ensemble (e.g. accessibility or compactness) or other structural propensities of the motif-containing domain (e.g. local structural propensities). Further, MYC2, MYC3 and MYC4 have been reported to interact with MYB TFs both from subgroup 12 (26) and from subgroup 19 (27). However, MYB TFs from subgroup 19 lack the MIM and did not interact in our system. Split-ubiquitin and similar techniques are strong for identifying new possible interactions and narrowing down interaction motifs or essential residues but, as all experimental methods, produce false positives and false negatives. Therefore, we applied complementary, orthogonal approaches to confirm the findings from our split-ubiquitin assays by showing biological function *in planta* (Figure 5) and *in vitro* interaction with a quantitative technique, bio-layer interferometry (Figure 6 and Supplementary Figure S7), demonstrating a correlation between binding affinity and activity. In this work, we identified two new MYC interacting MYB TFs, MYB95 and MYB47. Depending on the type of phylogenetic analysis, these MYB TFs end up close or distant to subgroup 12 (3,11). Our finding that they contain a functional MIM hints towards them being in a sister group to the subgroup 12 MYB TFs.

When characterising the MIM, we found slight differences between MYC3 and MYC4, as MYC4 appeared to be more promiscuous (Figure 4A; Supplementary Figures S5 and 6). This does not necessarily mean that MYC3 and MYC4 have different binding preferences but could be because MYC4 in general interacts stronger or is more abundant in the yeast cells, and therefore the interaction assayed by our split-ubiquitin system is more difficult to abolish. Most of the mutated versions we tested did not abolish binding, even when simultaneously mutating several conserved positively charged residues to negatively charged ones. These conserved residues were not critical for the interaction, but they might still contribute to interaction parameters, such as the affinity, kinetics or properties of the structural ensemble. Electrostatic interactions are known to be important drivers of long-range interactions that allow binding partners to locate each other but may not be crucial in our split-ubiquitin system since the cellular environment and abundance of the interacting proteins likely differs from the native situation in a plant cell.

### The evolution of short linear motifs can generate regulatory links

The MIM is a SLiM. SLiMs have distinctive features in terms of their interaction interfaces and evolution, compared to their counterparts in globular domain interactions and are common in intrinsically disordered regions of regulatory proteins and TFs (9,51,56). One unique feature of SLiMs as interaction modules, recently shown to be biologically relevant, is the possibility of displaying multiple copies of the same SLiM to use multivalent interactions for tuning activity of a functional complex (57). In MYB34, we observed a putative additional MIM, _197_LLNKMA_202_. The extra MIM displayed by MYB34 might allow multivalent interactions to fine-tune the transcriptional output of the TF complex.

Another distinctive feature of SLiMs is that they are much more likely to spontaneously and convergently evolve than globular interaction domains, predominantly because of their short length and presence in usually fast evolving disordered regions (51,55). This makes it challenging to assess the evolutionary relationships of proteins containing the same SLiMs (as we see for MYB95, MYB47 and MYB subgroup 12). The higher convergence of SLiMs increases the possibility of *de novo* interactions with signalling proteins, allowing signalling pathways to impact traits previously unaffected. The motif presented in this work mediates interaction with MYC2, MYC3 and MYC4, and could therefore, if evolving *de novo* in unrelated proteins, confer a new regulatory link to JA signalling.

The biologically active form of JA, JA-Ile, relieves JAZ-dependent repression of MYC TF activity by formation of a co-receptor complex of JAZ, JA-Ile and COI1, leading to ubiquitination and subsequent proteasomal degradation of JAZ (58–62). MYC2, MYC3 and MYC4 interact with JAZ repressor proteins through the JID in their N-terminal domains (34,58,61,62). The bHLH TFs GL3, EGL3 and TT8 also contain a JID in their N-terminal region; however, the JID present in GL3, EGL3 and TT8 is not involved in their interactions with JAZ protein, which are instead mediated through the C-terminal regions (63–65). These results, coupled with the finding that subgroup 12 MYB TFs interact with the JID of MYC2, MYC3 and MYC4 (26), but not with GL3, show that although there are sequence similarities between the N-terminal regions of the bHLH TFs from subgroups IIIe and IIIf, their molecular functions are not conserved. Further experiments are needed to determine the exact residues of MYC2, MYC3 and MYC4 involved in the interactions with subgroup 12 MYB TFs.

As MYB TFs from subgroup 12 require MYC interaction for activity, MYB TF activity in turn depends on the degradation of JAZ repressors to relieve MYC repression. The convergent evolution of a MIM in other proteins has the potential to establish a new regulatory connection linking protein activity to JA signalling. Spontaneous evolution of weakly binding, rudimentary motifs is possible and if the new interaction conferred is advantageous or deleterious, random mutagenesis and evolutionary pressure can quickly either fine-tune the specificity and affinity of the SLiM or abolish it (51). Yet, further studies are necessary to conclude whether evolution of a MIM in unrelated proteins is sufficient to connect protein activity to JA signalling through physical interactions with MYC TFs.

### Complex formation and competition

An interesting, but highly challenging question to address will be how big a role competition plays in determining functional output of the type of TF complexes discussed here. There is a high number of possible interactions (e.g. all six subgroup 12 MYB TFs are able to interact with MYC2, MYC3 and MYC4), but the output of the interactions is not the same (different classes of glucosinolates produced). Possibly, relative binding affinities and protein concentrations are fine-tuned to determine functional output of the complexes, relying also on the specificities of their DBDs and the resulting transcripts, possible post-translational modifications (PTMs) as well as yet other potential motifs present in the long disordered regions. Different classes of glucosinolates are not generally biosynthesized in the same cells (66). Further, MYC TFs have overlapping but not identical expression patterns (62,67), yet suggesting that competition could be relevant in cells where they co-occur. When we ectopically expressed *MYB29* we observed a negative correlation between the expression level and the accumulation of indole glucosinolates (Figure 5, Table 1 and Supplementary Table S1). Reduced levels of indole glucosinolates in lines ectopically expressing MYB29 have been reported before (23). Our data now indicate that this effect may arise as the MYB TFs controlling aliphatic and indole glucosinolate accumulation share the same MYC TF interaction partners, i.e. the indole glucosinolate-activating MYB TFs (MYB34, MYB51 and MYB122) may be outcompeted in their interaction with MYC2, MYC3 and MYC4 when overexpressing *MYB29*. This competition may only be relevant when competing genes are ectopically expressed, like here, as native expression patterns may prevent competing interactors from co-occurring, suppressing competition *in vivo*.

*In vivo* complex composition ultimately depends on the relative abundance of the complex components, their binding kinetics and their availability in the structural ensemble. Although all six subgroup 12 MYB TFs (and MYB95 and MYB47) interact with MYC2, MYC3 and MYC4 (26), differences in the MIM and flanking regions (Figure 3A) and different structural properties of the disordered regions harbouring the MIM, may result in different kinetics, leading to e.g. preferred partnerships considerably affecting complex formation and competition *in vivo*. To address how competition affects the phenotypic output of these TF complexes it will be necessary to determine binding affinities and kinetics of different MYB–MYC pairs, carry out competition assays, and achieve higher resolution of expression patterns, coupled with *in planta* validation.

### Functions of non-MYB regions in plant MYB TFs

Here, we provide an example of regulatory activity occurring outside the DBD of a plant MYB TF. The non-DBD regions of plant TFs contain extensive intrinsically disordered regions (6,7), and since disorder is so prevalent, it must be a feature selected for in evolution. As it has been demonstrated for other, mainly animal TFs, intrinsic disorder provides specific and unique functions to the proteins harbouring them, in particular, highly controlled regulatory functions (68–74). In these few recent examples of molecular functions provided by intrinsically disordered regions in TFs it was found that disorder (i) allows efficient binding between general transcriptional coactivators towards both their activators and repressors, and at the same time extremely efficient competition through complete displacement of activator by repressor (75), (ii) tunes activation and repression through ‘energetic frustration’, where intrinsically disordered domains can be allosterically coupled to other domains in terms of their structural ensembles (76) and (iii) tunes transcriptional output via multivalent binding to a negative regulator, through a disordered region, where both negative and positive cooperativity are at play, resulting in a gradient output of transcriptional activity (57). Although the functional importance of disorder is not well understood for plant MYB TFs, there are a number of studies linking the non-MYB regions of plant MYB TFs with regulatory PTMs (77–81), regulatory interactions (82–85) or their ability to activate or repress target genes (86–89).

Plant MYB subgroups are classified based on the presence of short, conserved sequence motifs, located in the non-MYB regions (3,11). These motifs have been useful for understanding phylogenetic relationships within the MYB family, and for inferring biological functions, as MYB TFs within the same subgroup often are involved in regulating the same or similar processes. However, the motifs are obviously not there to aid determination of evolutionary relationships but must be conserved because they confer molecular functions to the proteins harbouring them. This is the case for the motif defining subgroup 12, here identified as a SLiM crucial for interaction with MYC proteins. Given the high frequency of these conserved motifs in MYB TFs this implies that their disordered non-MYB regions mediate many interactions yet to be revealed, regulating their activity on the protein level in a subgroup-specific manner.

## Supplementary Material

gkz691_Supplemental_File
