# Supplementary material for: Specificity of MYB interactions relies on motifs in ordered and disordered contexts
Source: Nucleic Acids Res. 2019 Aug 10;47(18):9592–608. doi: 10.1093/nar/gkz691 (PMC6765112; doi:10.1093/nar/gkz691)
Supplement: gkz691_Supplemental_File [file gkz691_supplemental_file.pdf]

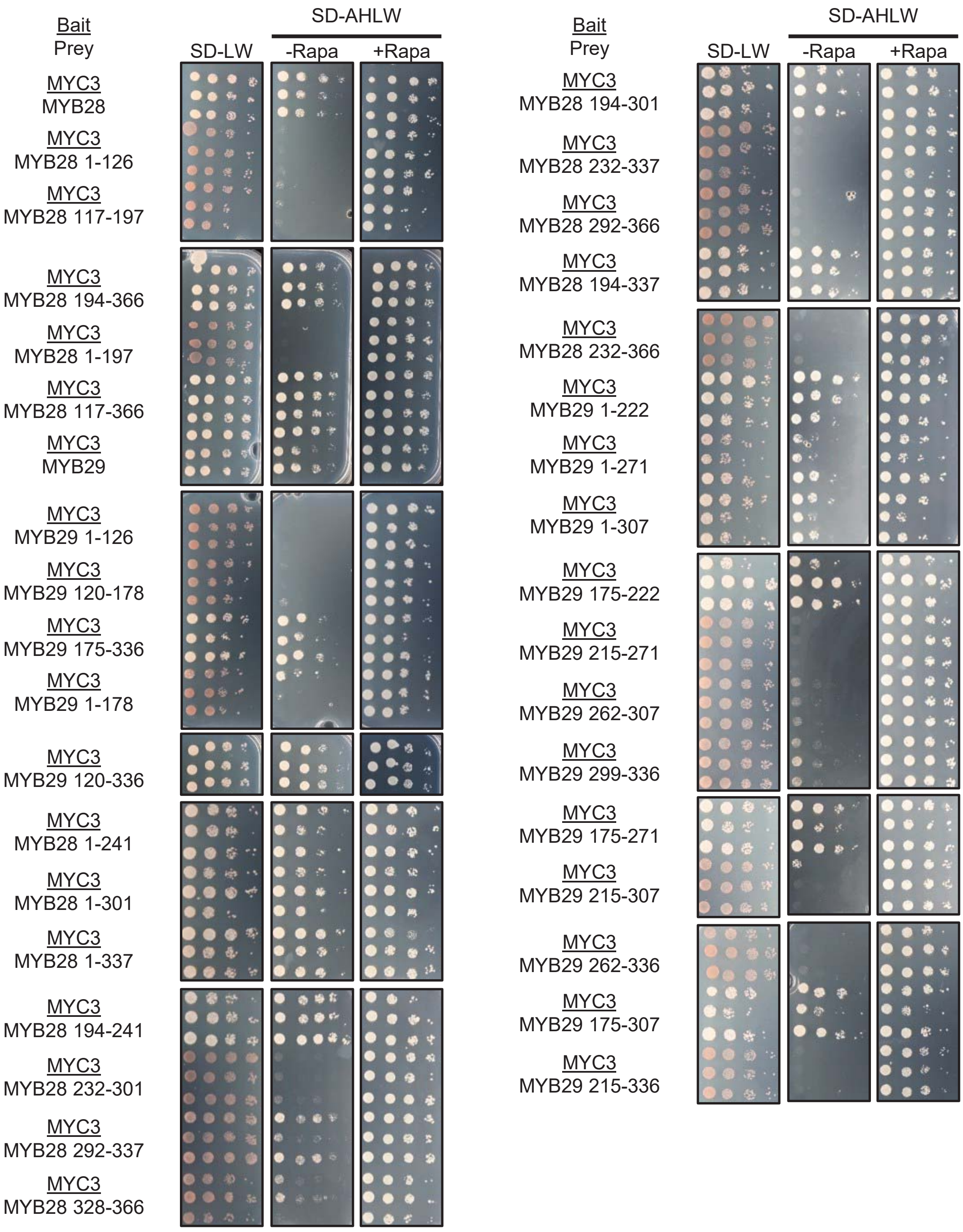

Supplementary figure 1. Split-ubiquitin assays between MYC3 (bait) against truncated versions of MYB28 and MYB29.

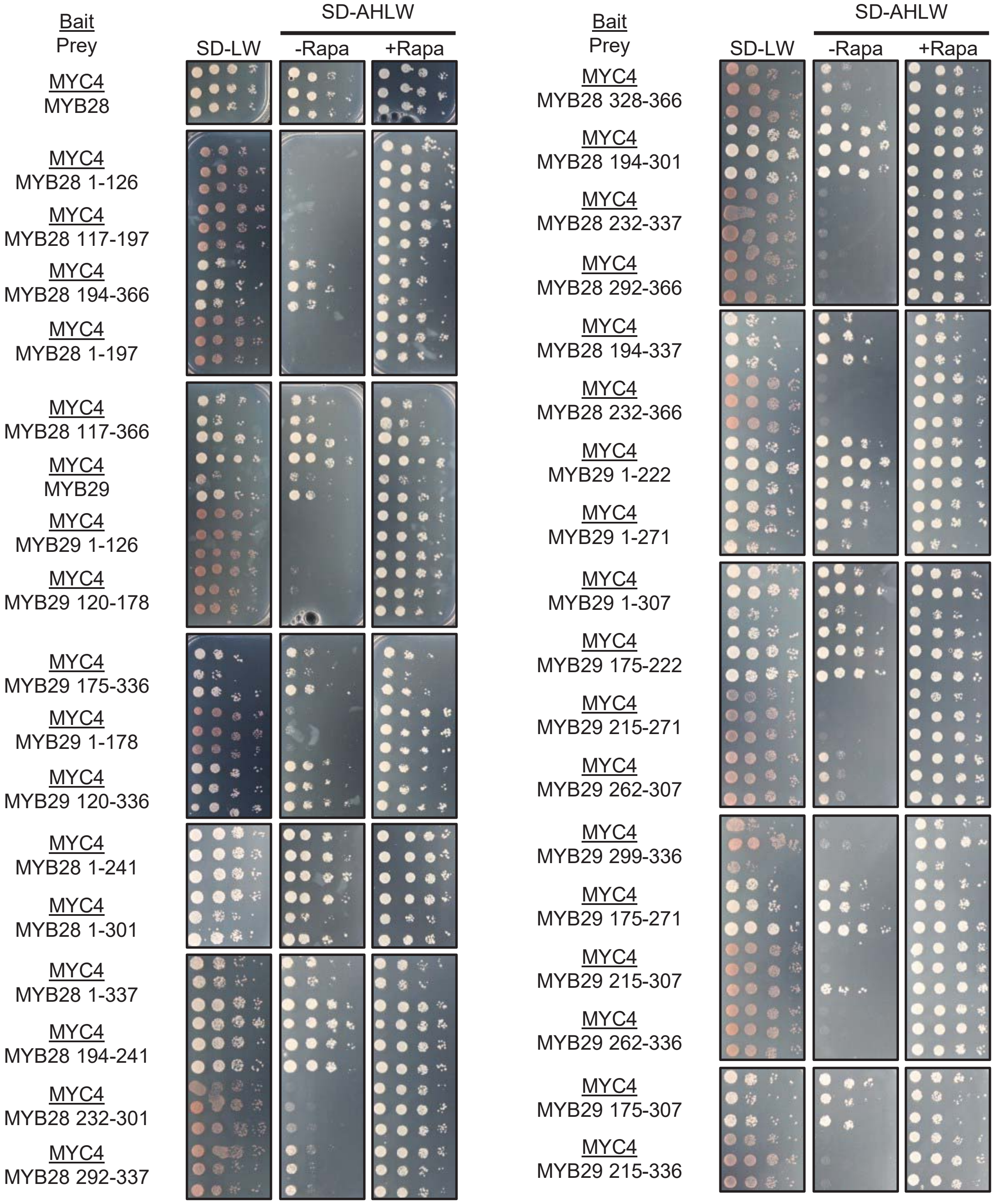

Supplementary figure 2. Split-ubiquitin assays between MYC4 (bait) against truncated versions of MYB28 and MYB29.

```
                          20                    40                    60
                           |                     |                     |
 MYB28 MSRKPCCVGE -GLKKGAWTT EEDKKLISYI HDHGEGGWRD IPQKAGLKRC GKSCRLRWTN 59
 MYB29 MSRKPCCVGE -GLKKGAWTA EEDKKLISYI HEHGEGGWRD IPQKAGLKRC GKSCRLRWAN 59
 MYB76 MSKRPYCIGE -GLKKGAWTT EEDKKLISYI HDHGEGGWRD IPEKAGLKRC GKSCRLRWTN 59
 MYB51 MVRTPCCKAE LGLKKGAWTP EEDQKLLSYL NRHGEGGWRT LPEKAGLKRC GKSCRLRWAN 60
MYB122 MVRTPCCRAE -GLKKGAWTQ EEDQKLIAYV QRHGEGGWRT LPDKAGLKRC GKSCRLRWAN 59
 MYB34 MVRTPCCKEE -GIKKGAWTP EEDQKLIAYL HLHGEGGWRT LPEKAGLKRC GKSCRLRWAN 59
 MYB75 MEGS-----S KGLRKGAWTT EEDSLLRQCI NKYGEGKWHQ VPVRAGLNRC RKSCRLRWLN 55
                          80                    100                   120
                           |                     |                     |
 MYB28 YLKPEIKRGE FSSEEEQIII MLHASRGNKW SVIARHLPRR TDNEIKNYWN THLKKRLMEQ 119
 MYB29 YLKPDIKRGE FSYEEEQIII MLHASRGNKW SVIARHLPKR TDNEIKNYWN THLKKLLIDK 119
 MYB76 YLKPDIKRGE FSYEEEQIII MLHASRGNKW SVIARHLPKR TDNEVKNYWN THLKKRLIDD 119
 MYB51 YLRPDIKRGE FTEDEERSII SLHALHGNKW SAIARGLPGR TDNEIKNYWN THIKKRLIKK 120
MYB122 YLRPDIKRGE FSQDEEDSII NLHAIHGNKW SAIARKIPRR TDNEIKNHWN THIKKCLVKK 119
 MYB34 YLRPDIKRGE FSPEEDDTII KLHALKGNKW AAIATSLAGR TDNEIKNYWN TNLKKRLKQK 119
 MYB75 YLKPSIKRGK LSSDEVDLLL RLHRLLGNRW SLIAGRLPGR TANDVKNYWN THLSKK---- 111
                          140                   160                   180
                           |                     |                     |
 MYB28 GIDPVTHKPL ASSS-NPTVD ENLNSPNASS SDKQYSRSSS MPFLSRPPPS SCNMVSKVSE 178
 MYB29 GIDPVTHKPL AYDS-NP--- ---------- -DEQ-SQSGS ISPKSLPPSS SKN----VPE 159
 MYB76 GIDPVTHKPL ASSNPNPVEP MKFDFQKKSN QDEHSSQSSS TTPASLPLSS NLN---SVKS 176
 MYB51 GIDPVTHKGI TSGT------ ---------- -------DKS ENL---PEKQ NVN------- 147
MYB122 GIDPLTHKSL LDGA------ ---------- -------GKS SDHSAHPEKS SV-------- 148
 MYB34 GIDAITHKPI -NST------ ---------- -------GQT ---------- ---------- 135
 MYB75 ------HEPC C--------- ---------- ---------- ---------- ---------- 116
                          200                   220                   240
                           |                     |                     |
 MYB28 LSSNDGTPIQ GSSLSCKKR- FKKSSSTSRL LNKVAAK-AT SIKDILSASM EGSL-SATTI 235
 MYB29 ITSSDETPKY DASLSSKKRC FKRSSSTSKL LNKVAAR-AS SMGTILGASI EGTLISSTPL 218
 MYB76 KISSGETQIE SGHVSCKKR- FGRSSSTSRL LNKVAAR-AS SIGNILSTSI EGTLRSPASS 234
 MYB51 -LTTSDHDLD NDKAKKNNKN F--GLSSASF LNKVANRFGK RINQSVLSEI IGSGGPLAST 204
MYB122 ------HDDK DDQNSNNKKL S--GSSSARF LNRVANRFGH RINHNVLSDI IGSNGLL--T 198
 MYB34 ---------- GFEPKVNKPV Y--SSGSARL LNRVASKYAV ELNRDLLTGI ISGNSTVA-- 181
 MYB75 ---------- --KIKMKKRD ITPIPTTPAL KNNVYKPRPR SF-------- ---------- 146
                          260                   280                   300
                           |                     |                     |
 MYB28 SHASFFNGFT EQIRNEEDSS NTSLTNTLAE FDPFSPSSLY PEHE------ IN-ATS---D 285
 MYB29 S--SCLN--- ------DDFS ETSQFQ-MEE FDPFYQSS-- -EHI------ IDHMKE---D 254
 MYB76 SGLP------ ---------- ---------- -DSFSQSY-- -EYM------ IDNKEDLGTS 258
 MYB51 SHTTNTTTTS VSVDSESVKS TS-------- -SSFAPTSNL LCHGTVATTP VSSNFDVDGN 255
MYB122 SHTTPTTSVS -----EGERS TS-------- -SSSTHTSSN L--------P INRSITVDAT 236
 MYB34 ---------- -----EDSQN SG-------- -DVDSPTSTL L--------- -NKMAATSVL 207
 MYB75 ---------- ------TVNN DCNHLNAPPK VDVNPPC--- ------LGLN INNVCDNSII 181
                          320                   340                   360
                           |                     |                     |
 MYB28 LNMDQ-DYDF SQ-------- FFEKFGGDNH NEENSMN--- ---------- -DLLMSDVSQ 322
 MYB29 ISINNSEYDF SQ-------- FLEQFSNNEG EEADNTGG-- -------GYN QDLLMSDVS- 296
 MYB76 IDLNIPEYDF PQ-------- FLEQLINDDD ENENIVGP-- ---------E QDLLMSDFP- 298
 MYB51 VNLTCSSSTF SDSSVNNPLM YCDNFVGNNN VDDEDTIGFS T-----FLND EDFMMLEESC 310
MYB122 ---SLSSSTF SDSP--DPCL Y-EEIVG--- -DIEDMTRFS SRCLSHVLSH EDLLMSVESC 286
 MYB34 INTTTTYSGF SDN-----CS FTDEF----- ---------- ----NEFFNN EE-ISDIYTT 242
 MYB75 YNKDKKKDQL VNNLIDGDNM WLEKFLEES- ---------- --------QE VDILVPEATT 222
                          380                   400
                           |                     |
 MYB28 EVSSTSVDDQ DNMVGNFEGW SNYLLDHTN- -----FMYDT DSDSLEKHF- --I 366
 MYB29 ---STSVDE- DEMMQNITGW SNYLLDHSD- -----FNYDT SQDYDDKNF- --I 336
 MYB76 ---STFVDE- DDILGDITSW STYLLDHPN- -----FMYES DQDSDEKNF- --L 338
 MYB51 VENTAFMKEL TRFLHEDEND ---VVD---- -VTPVYERQD LFDEIDNYFG --- 352
MYB122 LENTSFMREI TMIFQEDKIE ---TTSFNDS YVTPINEVDD SCEGIDNYFG --- 333
 MYB34 VDNFGFMEEL KSILSYGDAS AGVIENSPEV NVADAMEFID SWNEDDNMVG VFV 295
 MYB75 TEKGDTL--- ---------- ---------- ----AFDVDQ LWSLFDGETV KFD 248
```

Supplementary figure 3. Full-length alignment of MYB28, MYB29, MYB76, MYB51, MYB122, MYB34 and MYB75.

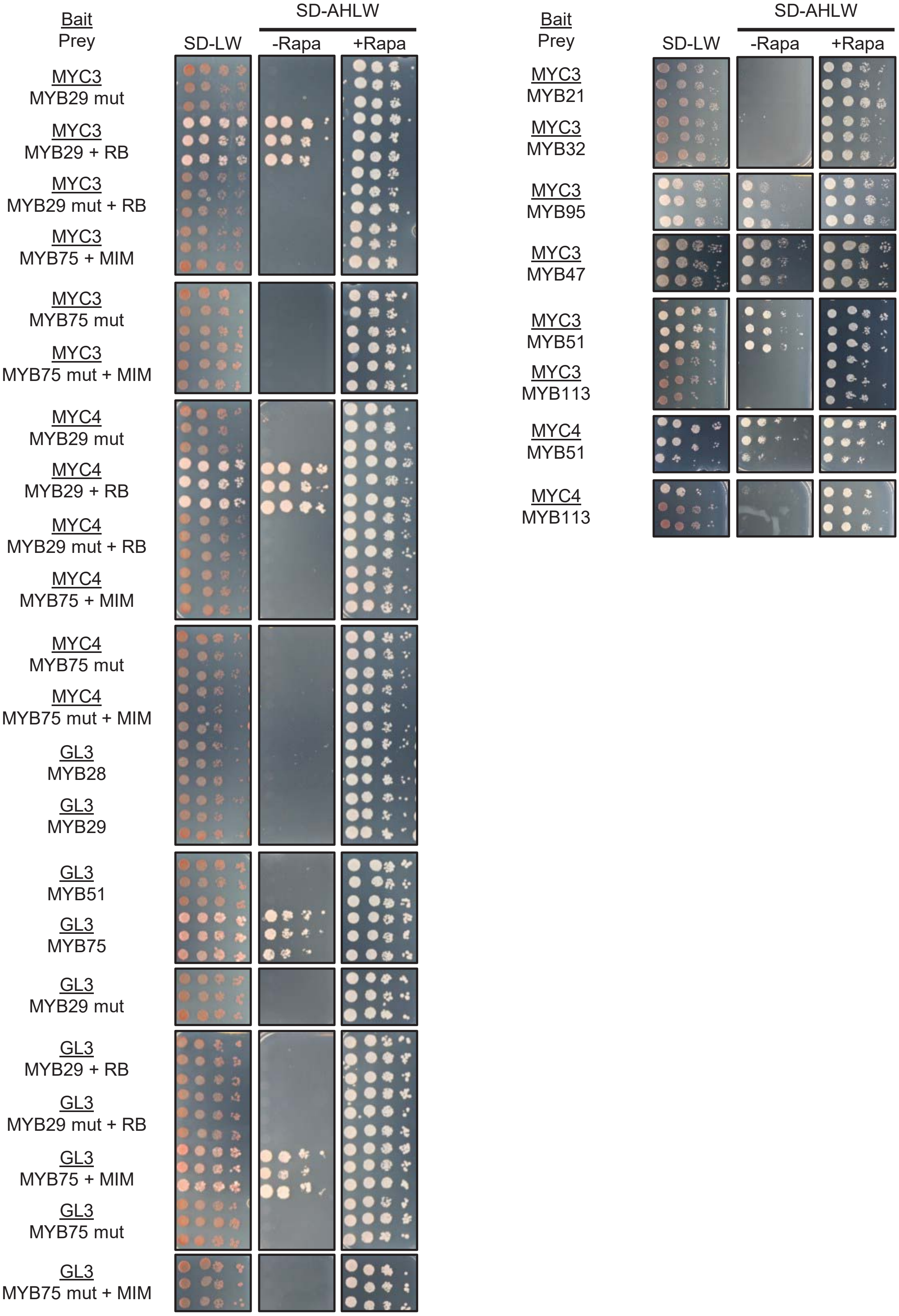

Supplementary figure 4. Split-ubiquitin assays between motif-swapped MYB29 and MYB75, and other full-length *A. thaliana* R2R3 MYB TFs against MYC3, MYC4 and GL3.

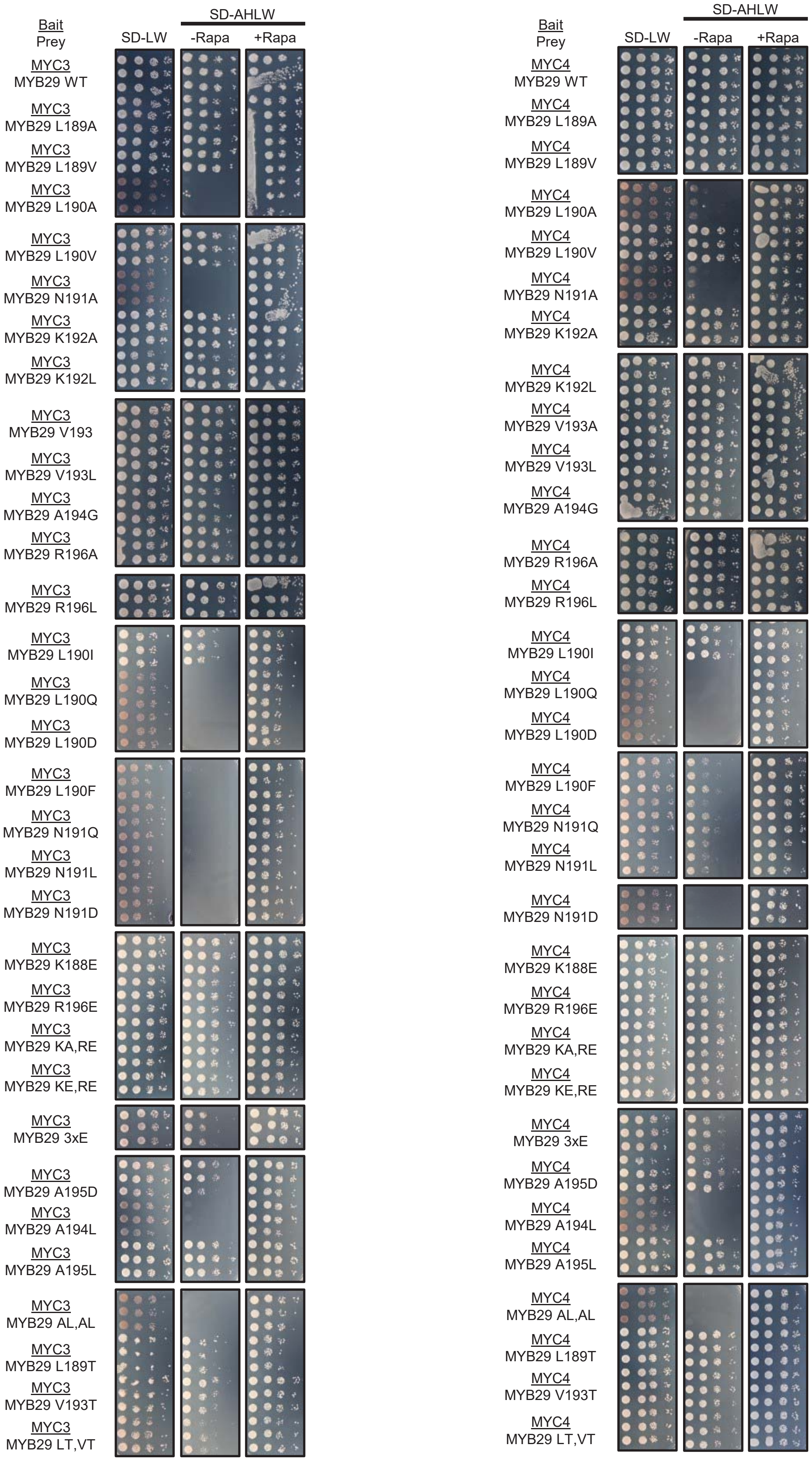

Supplementary figure 5. Split-ubiquitin assays between MYB29 (AA120-222) MIM-mutants against MYC3 and MYC4.

A

ID	MIM sequence	MYC3	MYC4
WT	-STSKLLNKVAARASS-	Yes	Yes
L189A	-STSKALNKVAARASS-	Yes	Yes
L189V	-STSKVLNKVAARASS-	Yes	Yes
L190A	-STSKLANKVAARASS-	**No**	**No**
L190V	-STSKLVNKVAARASS-	Yes	Yes
N191A	-STSKLLAKVAARASS-	**No**	**No**
K192A	-STSKLLNAVAARASS-	Yes	Yes
K192L	-STSKLLNLVAARASS-	Yes	Yes
V193A	-STSKLLNKAAARASS-	Yes	Yes
V193L	-STSKLLNKLAARASS-	Yes	Yes
A194G	-STSKLLNKVGARASS-	Yes	Yes
R196A	-STSKLLNKVAAAASS-	Yes	Yes
R196L	-STSKLLNKVAALASS-	Yes	Yes

B

ID	MIM sequence	MYC3	MYC4
L190I	-STSKLINKVAARASS-	Yes	Yes
L190Q	-STSKLQNKVAARASS-	**No**	**No**
L190D	-STSKLDNKVAARASS-	**No**	**No**
L190F	-STSKLFNKVAARASS-	**No**	(Yes)
N191Q	-STSKLLQKVAARASS-	**No**	(Yes)
N191L	-STSKLLLKVAARASS-	**No**	(Yes)
N191D	-STSKLLDKVAARASS-	**No**	**No**

C

ID	MIM sequence	MYC3	MYC4
K188E	-STSELLNKVAARASS-	Yes	Yes
R196E	-STSKLLNKVAAEASS-	Yes	Yes
KA,RE	-STSALLNKVAAEASS-	Yes	Yes
KE,RE	-STSELLNKVAAEASS-	Yes	Yes
3xE	-STSELLNEVAAEASS-	(Yes)	Yes

D

ID	MIM sequence	MYC3	MYC4
A194L	-STSKLLNKVLARASS-	**No**	**No**
A195L	-STSKLLNKVALRASS-	Yes	Yes
AL,AL	-STSKLLNKVLLRASS-	**No**	**No**
A195D	-STSKLLNKVADRASS-	Yes	Yes
L189T	-STSKTLNKVAARASS-	Yes	Yes
V193T	-STSKLLNKTAARASS-	Yes	Yes
LT,VT	-STSKTLNKTAARASS-	Yes	Yes

E

ID	pI
WT	11.4
K188E	9.8
R196E	9.6
KA,RE	6.9
KE,RE	4.6
3xE	3.2

Supplementary figure 6. Characterisation of MIM. (A-D) Results from split-ubiquitin assays of all the mutated versions of MYB29 (AAs 120-222) tested against MYC3 and MYC4. ‘Yes’ indicates interaction, ‘No’ indicates lack of interaction, while ‘(Yes)’ is a clear, but possibly weaker interaction than ‘Yes’, (see e.g. MYC3 against 3xE in supplementary figure 5). (E) Isoelectric point (pI) of some of the 15 AA stretches shown in (A-D) calculated using CLC Main Workbench.

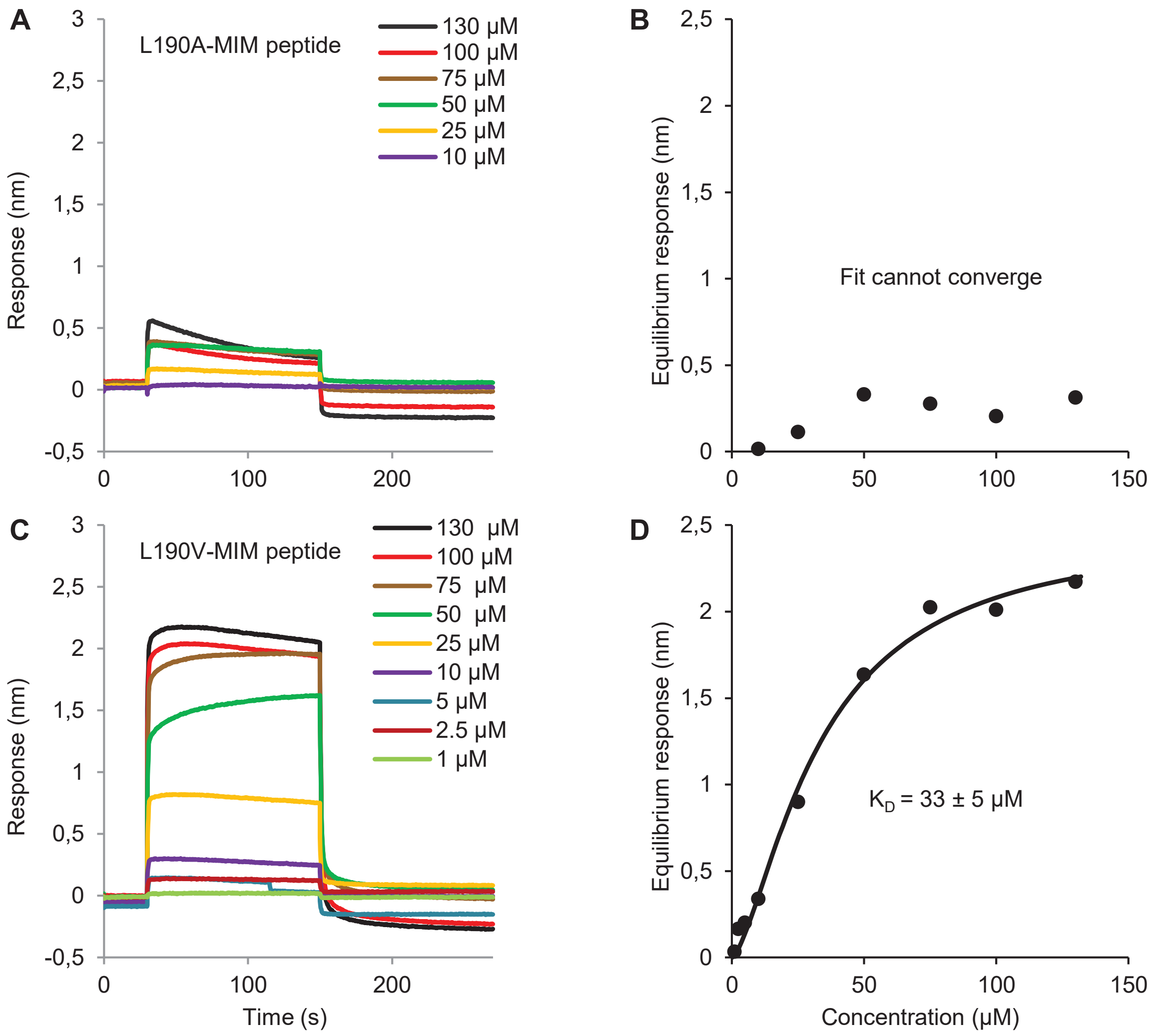

Supplementary figure 7. *In vitro* interaction between mutated MIM peptides and MYC4Nt. N-terminally biotinylated 22 residue peptides comprising the L190A (A-B) or L190V (C-D) MIM sequence of MYB29 were immobilized on streptavidin biosensors, and the binding of a dilution series of MYC4Nt was detected. (A) and (C) Referenced sensorgrams. (B) and (D) Fitting of equilibrium responses to a Hill equation, including the equilibrium dissociation constant (K_D) ± standard error of the fit.

Supplementary Table 1. Glucosinolate analysis of Col-0, *myb29-1*, or *myb29-1* plants expressing *pro35S:MYB29-L190A*, *pro35S:MYB29-L190V* or *pro35S:MYB29-WT*.

Short chain aliphatic glucosinolates

Factor	Df	Sum Sq	Mean Sq	F value	Pr(>F)	
Genotype	4	1.903	0.4757	14.38	6.47e-09	***
Relative MYB29 expression	1	1.085	1.0849	32.81	1.67e-07	***
Genotype x MYB29 expression	4	3.628	0.9069	27.42	2.12e-14	***
Residuals	81	2.679	0.0331			

Long chain aliphatic glucosinolates

Factor	Df	Sum Sq	Mean Sq	F value	Pr(>F)	
Genotype	4	0.5965	0.14913	5.275	0.000794	***
Relative MYB29 expression	1	0.0453	0.04526	1.601	0.209410	
Genotype x MYB29 expression	4	0.0403	0.01007	0.356	0.838986	
Residuals	81	2.2900	0.02827			

Indole glucosinolates

Factor	Df	Sum Sq	Mean Sq	F value	Pr(>F)	
Genotype	4	0.1752	0.04379	4.991	0.001201	**
Relative MYB29 expression	1	0.1344	0.13444	15.322	0.000188	***
Genotype x MYB29 expression	4	0.2586	0.06466	7.369	4.1e-05	***
Residuals	81	0.7107	0.00877			

Leaf glucosinolates were analysed and qPCR performed on transgenic T1 plants positive for the selection marker (or grown along Col-0 or *myb29-1* plants). 17-19 plants of each genotype/construct combination were analysed. Significant differences between short chain aliphatic glucosinolates, long chain aliphatic glucosinolates, and indole glucosinolates were tested with an ANCOVA model with the factors: Genotype/construct combination, relative *MYB29* expression, and the interaction between genotype/construct and relative *MYB29* expression. Significance codes: P<0.001***, P<0.01**, P<0.05*.

Supplementary Table 2. Individual leaf glucosinolate levels in Col-0, *myb29-1*, or *myb29-1* plants expressing *pro35S:MYB29-L190A*, *pro35S:MYB29-L190V* or *pro35S:MYB29-WT* shown in pmol/mg fresh weight ± s.e.m. Short chain aliphatic glucosinolates: 3-methylthiopropyl, 3-methylsulfinylpropyl, 4-methylthiobutyl, 4-methylsulfinylbutyl, 5-methylsulfinylpentyl. Long chain aliphatic glucosinolates: 7-methylthioheptyl, 7-methylsulfinylheptyl, 8-methylthiooctyl, 8-methylsulfinyloctyl. Indole glucosinolates: indol-3-ylmethyl, 4-methoxyindol-3-ylmethyl, N-methoxyindol-3-ylmethyl.

Genotype<br>*pro35S*	Col-0<br>-	*myb29-1*<br>-	*myb29-1*<br>*MYB29-L190A*	*myb29-1*<br>*MYB29-L190V*	*myb29-1*<br>*MYB29-WT*
n	18	18	17	19	19
3-methylthiopropyl	2.3 ± 0.3	0.6 ± 0.1	0.5 ± 0.1	0.1 ± 0.1	0.1 ± 0.0
3-methylsulfinylpropyl	26.0 ± 2.2	5.3 ± 0.6	5.5 ± 0.6	11.7 ± 2.4	26.7 ± 7.2
4-methylthiobutyl	164.1 ± 20.1	48.2 ± 7.4	31.7 ± 4.0	11.5 ± 2.4	8.5 ± 2.0
4-methylsulfinylbutyl	222.1 ± 20.9	56.9 ± 6.5	66.7 ± 7.8	170.4 ± 36.3	318.3 ± 100.7
5-methylsulfinylpentyl	35.2 ± 2.6	10.9 ± 0.9	14.3 ± 1.6	47.3 ± 10.9	82.8 ± 26.1
7-methylthioheptyl	12.1 ± 1.4	11.1 ± 1.1	10.8 ± 1.3	3.5 ± 0.6	4.4 ± 0.8
7-methylsulfinylheptyl	47.8 ± 4.9	25.3 ± 2.3	29.6 ± 4.0	19.0 ± 2.8	34.4 ± 10.6
8-methylthiooctyl	19.0 ± 2.9	19.7 ± 3.0	15.3 ± 2.1	5.2 ± 1.1	5.8 ± 1.3
8-methylsulfinyloctyl	306.7 ± 37.1	197.2 ± 25.6	224.5 ± 32.2	121.9 ± 23.2	149.8 ± 42.1
indol-3-ylmethyl	334.6 ± 16.9	361.3 ± 21.4	357.4 ± 19.0	294.6 ± 24.0	251.2 ± 38.4
4-methoxyindol-3-ylmethyl	26.5 ± 2.2	20.1 ± 0.9	18.8 ± 1.1	16.0 ± 1.1	17.3 ± 1.1
N-methoxyindol-3-ylmethyl	20.4 ± 2.4	17.3 ± 1.7	17.9 ± 1.8	19.1 ± 2.5	17.5 ± 1.6
2-phenylethyl	19.6 ± 1.7	7.0 ± 0.8	9.8 ± 1.2	25.0 ± 5.1	46.5 ± 17.1
